# Emerging Therapeutics for Immune Tolerance: Tolerogenic Vaccines, T cell Therapy, and IL-2 Therapy

**DOI:** 10.3389/fimmu.2021.657768

**Published:** 2021-03-29

**Authors:** Cody D. Moorman, Sue J. Sohn, Hyewon Phee

**Affiliations:** Department of Inflammation and Oncology, Amgen Research, Amgen Inc., South San Francisco, CA, United States

**Keywords:** tolerogenic vaccine, autoimmune disease, CAR-Tregs, IL-2 mutein, low-dose IL-2 therapy, antigen-specific tolerance, immune tolerance

## Abstract

Autoimmune diseases affect roughly 5-10% of the total population, with women affected more than men. The standard treatment for autoimmune or autoinflammatory diseases had long been immunosuppressive agents until the advent of immunomodulatory biologic drugs, which aimed at blocking inflammatory mediators, including proinflammatory cytokines. At the frontier of these biologic drugs are TNF-α blockers. These therapies inhibit the proinflammatory action of TNF-α in common autoimmune diseases such as rheumatoid arthritis, psoriasis, ulcerative colitis, and Crohn’s disease. TNF-α blockade quickly became the “standard of care” for these autoimmune diseases due to their effectiveness in controlling disease and decreasing patient’s adverse risk profiles compared to broad-spectrum immunosuppressive agents. However, anti-TNF-α therapies have limitations, including known adverse safety risk, loss of therapeutic efficacy due to drug resistance, and lack of efficacy in numerous autoimmune diseases, including multiple sclerosis. The next wave of truly transformative therapeutics should aspire to provide a cure by selectively suppressing pathogenic autoantigen-specific immune responses while leaving the rest of the immune system intact to control infectious diseases and malignancies. In this review, we will focus on three main areas of active research in immune tolerance. First, tolerogenic vaccines aiming at robust, lasting autoantigen-specific immune tolerance. Second, T cell therapies using Tregs (either polyclonal, antigen-specific, or genetically engineered to express chimeric antigen receptors) to establish active dominant immune tolerance or T cells (engineered to express chimeric antigen receptors) to delete pathogenic immune cells. Third, IL-2 therapies aiming at expanding immunosuppressive regulatory T cells *in vivo*.

## Introduction

The mammalian immune system evolved to protect our bodies from foreign pathogens and intrinsic aberrant malignancies while concurrently preventing deleterious immune responses toward self (1). Immune tolerance co-evolved as a safety system that maintains a state of immune unresponsiveness to autoantigens and self-tissues (2, 3). There are two mechanisms that maintain immunological tolerance denominated central and peripheral tolerance. Central tolerance occurs during lymphocyte development in the primary lymphoid organs (i.e. thymus and bone marrow), where T or B cell clones that recognize autoantigens with high-affinity are deleted. Peripheral tolerance evolved to counteract autoantigen-recognizing T or B cells that escape central tolerance. Peripheral tolerance occurs in the secondary lymphoid organs (e.g. spleen, lymph nodes, and mucosal/gut associated lymphoid tissues) and peripheral tissues. Mechanisms of peripheral tolerance include inactivation of autoantigen-recognizing T and B cells by the induction of apoptosis, anergy or conversion into immunosuppressive regulatory cells. In addition, suppressor immune cells such as FOXP3^+^ regulatory T cells (Tregs) exert dominant immune suppression to control autoreactive T and B cells. Evidence suggest that a patient’s genetic predisposition together with environmental factors, such as exposure to pathogens that exhibit molecular mimicry, disturb immune tolerance (4). Loss of immune tolerance to autoantigens associated with a specific organ results in the activation of organ-specific T and B cells that in turn cause organ-specific inflammation and the development of autoimmune diseases such as multiple sclerosis (MS) (5), rheumatoid arthritis (RA) (6), psoriasis (7), and type 1 diabetes (T1D) (8). Thus, therapeutics that induce, restore, and maintain immune tolerance toward these autoantigens represent the “Holy Grail” of treatments for autoimmune diseases.

For the last two decades, our understanding of immunology exploded with the advent of technologies that allowed high throughput screening and generation of large molecule biologics such as monoclonal antibodies (mAb) and small molecule compounds. As an outcome, there was a huge success of anti-TNF-α blockers, which became the “standard of care” for many autoimmune diseases (9). The success of anti-TNF-α blockers stimulated the development of large molecule biologics that block the function of various cytokines (i.e. IL-1, IL-6, IL-12, IL-17, and IL-23) (10, 11) and small molecule compounds that inhibit molecular interactions involved in the generation of inflammation (i.e. JAKs, TYK2, IRAK4, BTK, SYK, RIPs, and TPL2) (12–14).

Despite the success of anti-TNF-α blockers and other immunomodulatory therapies, there are still significant unmet clinical needs. First, currently approved therapies are immunomodulatory and provide a remedy to relieve symptoms but do not provide a cure by directly addressing the loss of immune tolerance. Second, because most of these therapeutics work by reducing systemic inflammation, they have numerous adverse safety risks including increased susceptibility to opportunistic infections or malignancies and have detrimental side effects (15–17). Third, these agents lack efficacy in refractory autoimmune diseases (18) and in patients that are or become unresponsive to treatment (19).

Novel therapeutics are emerging to achieve immune tolerance in autoimmunity. Most of these novel therapeutics harness known immune tolerance mechanisms, such as increasing FOXP3^+^ Tregs and inducing anergy or deletion of pathologic immune cells. A subset of these therapeutics includes coinhibitory checkpoint agonists and costimulatory checkpoint antagonists to expand Tregs and/or dampening pathogenic effector cells. However, these therapies are beyond the scope of this review, but are discussed in multiple outstanding reviews (20–23). In this review, we will focus on three main categories of therapeutics that drive immune tolerance. First, tolerogenic vaccines designed to elicit autoantigen-specific immune tolerance. Second, T cell therapies using Tregs to establish active dominant immune tolerance or T cells to delete pathogenic immune cells for the treatment of autoimmunity. Third, IL-2 based therapies to expand immunosuppressive Tregs.

## Tolerogenic Vaccines for the Induction of Antigen-Specific Tolerance

### Antigen-Specific Immune Tolerance

Risks associated systemic immune suppression or immunomodulatory therapies for the treatment of autoimmune diseases could be reduced or even halted if antigen-specific tolerance was generated. Unlike general immune suppression, antigen-specific tolerance inhibits the pathogenic autoantigen-specific immune responses that drive autoimmune diseases, while leaving the rest of the immune system intact. Therefore, antigen-specific tolerance is the logical next step in treating autoimmune diseases. Among the emerging therapeutics, tolerogenic vaccines are gaining noticeable traction (24–28). Despite the potentials of antigen-specific tolerance, there are at least two outstanding questions to resolve. First, although knowledge of which autoantigens are associated with specific autoimmune diseases continues to grow, most of them are still not known (29–31). Second, because it is unclear how many autoantigens are involved in individual autoimmune diseases, it is still uncertain whether tolerance toward one or few autoantigens can reverse autoimmunity. Therefore, therapies that not only drive antigen-specific but more extensive organ-specific tolerance would likely have the greatest efficacy in a clinical setting. Here we discuss novel therapeutics that derive antigen-specific and/or organ-specific tolerance.

#### CD4^+^ FOXP3^+^ Tregs as the Master Regulators of Immune Tolerance

Immunosuppressive CD4^+^ FOXP3^+^ Tregs are required for immune tolerance. Genetic deficiencies of the transcription factor *Foxp3*, the Treg master regulator, results in the fatal systemic autoimmune diseases, immunodysregulation polyendocrinopathy enteropathy X-linked (IPEX) in humans and Scurfy in mice (32–34). CD4^+^ FOXP3^+^ Tregs comprise approximately 4-12% of the peripheral CD4^+^ T cell population and are identified based on the expression of FOXP3, high levels of CD25 and low levels of CD127 (35). FOXP3 controls the transcriptional programing of Tregs and imparts an immunosuppressive phenotype because experimental ectopic expression of FOXP3 in T cells (36–39), B cells (40), and myeloid cells (41) confers immunosuppressive capabilities. Likewise, genetic disruption or downregulation of FOXP3 impairs immunosuppressive capabilities and renders Tregs immunostimulatory (42, 43). CD4^+^ FOXP3^+^ Tregs have a T cell receptor (TCR) repertoire that is skewed toward the recognition of self-antigens (44). The self-reactive TCR directs Treg trafficking to self-tissues where cognate autoantigens are presented, resulting in TCR engagement and immunosuppressive effector functions (24). Tregs require TCR activation *via* their cognate antigen (45, 46) and the cytokine IL-2 (47) to suppress multiple facets of the immune system including T conventional cells (Tcons), B cells, and myeloid cells (48, 49). For example, when activated FOXP3^+^ Tregs express the immunosuppressive cytokines IL-10, IL-35, and TGF-β that inhibit Tcon and DC activation (50); suppress antigen presenting cells (APCs) expression of antigen presentation molecules MHCI and MHCII, costimulatory molecules CD80, CD86, and CD40 and proinflammatory cytokines IL-12 and IL-6 as well as differentiate dendritic cells (DC) into tolerogenic DCs (tDCs) (51–54); express the ectoenzymes, CD39 and CD72, which catabolize proinflammatory extracellular ATP/ADP into anti-inflammatory AMP (55); express the inhibitory receptors CTLA-4, LAG-3, PD1, TIGIT, GITR, and TIM-3 to block APC maturation and T cell activation (56); produce the cytotoxic molecules Galectin-9, Fas-L, TRAIL, Perforin, and Granzyme-B to kill effector T cells and inflammatory APCs (57); sequester IL-2 to inhibit Tcon access to this critical cytokine required for T cell proliferation, function, and survival (58, 59); and finally, deplete local glucose disrupting the metabolic needs of effector T cells (60). These FOXP3^+^ Treg effector functions create an immunosuppressive microenvironment at the site of autoantigen recognition preventing autoimmune responses. Because FOXP3^+^ Tregs play a fundamental role in tolerance, it is crucial that Tregs maintain phenotypical and functional stability in both quiescent and inflammatory environment associated with autoimmune disease. Of the major mechanisms that control Treg stability, demethylation of the Treg-specific demethylated region (TSDR), low-moderate TCR-antigen recognition efficiency, and IL-2 signaling are among the most prominent signals that maintain Treg stability. There are several informative reviews that provide an in-depth review on Treg stability (24, 61–63).

Interestingly, activated antigen-specific Tregs can also maintain tolerance to antigens beyond their cognate antigen specificity *via* regulatory mechanisms termed, bystander or linked suppression and infectious tolerance (64). Activated Tregs employ numerous effector functions to create an immunosuppressive microenvironment that can suppresses and/or tolerizes local T cells with alternative antigen specificities. This indiscriminate local suppression has been termed “linked/bystander” suppression because both the Treg- and Tcon-cognate antigens must be spatially colocalized and presented on the same APC. Simply, a Treg specific for antigen-X can suppress a Tcon specific for antigen-Y when both antigens X and Y are presented on the same APC (65).

Furthermore, Tregs can induce T cells to differentiate into regulatory T cell subsets. This conversion requires spatial colocalization and coactivation of both the FOXP3^+^ Treg and T cell. The recruitment of T cells into regulatory T cell subsets has been termed “infectious tolerance” because the new regulatory T cells can maintain tolerance independently of the original stimuli thereby spreading tolerance. Simply, a Treg specific for antigen-X can induce a T cell specific for antigen-Y to become a regulatory T cell when both X and Y are presented on the same APC. The antigen-Y-specific regulatory T cell can then mediate active dominant antigen-specific tolerance for antigen-Y when antigen-X is no longer present (24). For example, FOXP3^+^ Tregs express TGF-β, IL-10, and IL-35, that in turn differentiate T cells into FOXP3^+^ Tregs, type 1 regulatory T cells (Tr1), and inducible IL-35 producing regulatory T cells (iTr35), respectively (66–70). Likewise, Tr1 and iTr35 can mediate infectious tolerance (67, 71).

In the context of immune tolerance, therapeutics that elicit Treg responses mediating linked/bystander suppression and infectious tolerance toward organ-specific autoantigens would be ideal to control organ-specific autoimmune disease that involve numerous or unidentified autoantigens. Linked/bystander suppression and infectious tolerance have beneficial roles in autoimmunity, allergy, and organ transplant, while also having a detrimental effect on the clearance of cancers and pathogens (72). It is difficult to experimentally differentiate linked/bystander suppression from infectious tolerance; therefore, we will collectively term them bystander suppression.

#### Dendritic Cells (DCs) as a Key Cell Type Governing Immune Tolerance

DCs are a subset of professional APCs that facilitate T cell responses to generate both protective immune responses toward pathogens/cancer and tolerant immune responses toward self-antigens (73). DCs are the key governing APC of immune tolerance because depletion of CD11c^+^ DCs resulted in the development of fatal autoimmunity in mice (74). DCs capture, processes, and present antigens while integrating environmental signals, often supplied by T cells (e.g. CD40L or PD1), to modulate the expression of stimulatory and inhibitory molecules to direct T cell responses (73). DCs are a functionally and phenotypically heterologous group of APCs. DCs retain an “immature” phenotype during homeostatic conditions, characterized by minimal expression of co-stimulatory molecules and proinflammatory cytokines. Environmental cues such as pathogen- or danger- associated molecular pattern molecules and co-stimulatory molecules direct immature DCs to differentiate into mature DCs, that upregulate the expression of co-stimulatory molecules and proinflammatory cytokines. These mature DCs, in turn, provide antigen presentation, co-stimulation, and cytokine help, activating effector Tcons to generate protective adaptive immunity (73).

A third subset of “semi-immature” DCs exhibit an intermediate maturation status and may function as tolerogenic DCs (tDCs) (75). Tolerogenic DCs lack a defined transcriptional regulator and instead are classified by their common phenotypical and functional attributes. Typically, tDCs express low levels of MHCI/MHCII, co-stimulatory molecules, and inflammatory cytokines. In addition, tDCs express a unique transcriptional program that results in the expression of immunosuppressive molecules such as nitric oxide, indoleamine 2,3-dioxygenase, anti-inflammatory cytokines IL-10 and TGF-β, and inhibitory co-receptors PD-L1/2, ICOSL, B7-H4, and B7-H3 (73).

How do DCs maintain tolerance? The two pillars of DC mediated tolerance are the induction of apoptosis or anergy of autoreactive T cells and induction, expansion, and maintenance of Tregs. During homeostatic conditions, immature DCs present self-antigens in the absence of extensive co-stimulation or cytokine help, leading to unproductive T cell activation and autoreactive T cell apoptosis and anergy (73, 76). Because tDCs do not express high levels of co-stimulatory receptors or inflammatory mediators, but rather express high levels of inhibitory co-receptors and immunosuppressive molecules, tDC can induce T cell anergy or death under inflammatory conditions. Another way DCs control autoimmune diseases is by expanding and maintain the Treg pool. For example, Treg populations are dependent on DCs because, depletion of CD11c^+^ DCs reduced Treg populations while increasing DC numbers expanded Treg populations in mice (77, 78). Moreover, DCs support Treg function because Tregs from DC-depleted mice had reduced suppressive capabilities (79). DCs expand Tregs when MHCII-driven autoantigen presentation is paired with immunosuppressive molecules such IL-10, IDO, PGE-2, and TGF-β. Even under inflammatory conditions, DCs expressing autoantigens can expand autoantigen-specific Tregs (79). While there is evidence that both immature and mature DCs can support Treg induction, it is still unclear which subtype of DC confers tolerogenic responses under different circumstances.

Beyond DCs, there are other MHCII expressing APCs that provoke tolerance. These cells include liver sinusoidal endothelial cells (LSECs), macrophages, and T cells. LSECs express MCHII, low levels of costimulatory molecules and do not produce the proinflammatory cytokine IL-12 and thus fail to stimulate pathogenic Th1 responses. Instead, LSEC expand immunosuppressive Tregs *via* the expression of surface bound TGF-β and Jagged family of Notch ligands (80, 81). Immunosuppressive macrophages such as alternatively activated macrophages (AAMs)/M2-like macrophages, tumor associated macrophages, and marginal zone macrophages produce high levels of IL-10, TGF-β and PD-L1 to promote the expansion and differentiation of Tregs or deletion of autoreactive T cell (82–85). T cells can also express MHCII and favor tolerance *via* the induction of T cell anergy and apoptosis (86).

### Antigen-Specific Tolerogenic Vaccines Platforms

Because immune tolerance is orchestrated by APCs, numerous tolerogenic vaccine platforms have been developed to deliver autoantigens to specific APC subtypes. Some of these tolerogenic vaccine platforms include protein/peptide-, nanoparticle-, and DNA/RNA-based vaccines. Furthermore, immunosuppressive cell types such as tDCs, have been manipulated and expanded *ex vivo* and reintroduced as cell-based tolerogenic vaccines. Together, these agents can be summarized as antigen-specific tolerogenic vaccines.

Due to the breath of tolerogenic vaccines being investigated and broad number of preclinical autoimmune disease models, it is practical to compare different tolerogenic vaccine platforms within the contexts of a single disease model. Thus, for this review, we will focus on tolerogenic vaccines tested in preclinical models of experimental autoimmune encephalomyelitis (EAE), the animal model for MS. EAE models have been extensively utilized as a preclinical animal model for the development of tolerogenic vaccines. EAE is a CD4^+^ T cell driven CNS-specific autoimmune disease that involves complex immune responses and numerous neuroantigen targets including myelin oligodendrocyte glycoprotein (MOG), myelin basic protein (MBP) and proteolipid protein (PLP), that can mimic some of the complexities of human autoimmune diseases but with limitations. As with any preclinical model, EAE has many differences in comparison to that pathogenesis of MS. Thus, readers should take the results of preclinical EAE studies to understand the mechanism of tolerance, rather than to gauge potential therapeutic efficacy in patients.

#### Peptide- and Protein-Based Vaccines

In preclinical studies, administration of naked myelin peptides under quiescent homeostatic conditions by various routes including intravenous (IV), subcutaneous (SC), epicutaneous, intraperitoneal (IP), intranasal, intrathymic, or oral induced antigen-specific tolerance and suppressed EAE (87–93). However, when included in the immunogenic complete Freund’s adjuvant (CFA), these same naked myelin peptides provoked encephalitogenic priming and the active induction of EAE (94). Therefore, strategies were devised to induce antigen-specific tolerance in proinflammatory environments during active EAE. These strategies included targeting autoantigens to immunologic niches that favor tolerance such as the skin and oral mucosa (95, 96); administering high doses of peptide IV to induce effector Tcon apoptosis (97); and modifying myelin peptides by altering the amino acid sequence (altered peptide ligands) to decrease TCR-antigen recognition efficiency and induce regulatory T cells and/or effector T cell anergy and apoptosis (98).

Several of these strategies were advanced into human clinical trials (**Table 1**). The first of these was a phase I clinical trial testing daily oral administration of encapsulated bovine myelin in patients with relapsing-remitting MS (99). It was determined that oral bovine myelin was safe and reduced the frequency of MBP-specific T cells; however, in a larger phase III clinical trial, oral bovine myelin did not significantly improve MS (113). Subsequently, phase I and II clinical trials tested the safety and efficacy of “ATX-MS-1467” which was comprised of MBP_30-44_, MBP_131-145_, MBP_140-154_, and MBP_83-99_ and was administered ID or SC to patients with relapsing-remitting or secondary progressive MS (100). The therapy was safe and resulted in a significant decrease of new/persisting CNS lesions. In addition, a phase I clinical trial examined the safety of a peptide vaccine comprised of MBP_85-99_, MOG_35-55_, and PLP_139-155_ that was administered *via* skin patch to patients with relapsing-remitting MS (102). The therapy was safe and resulted in a significant reduction of CNS lesions and reduced clinical disease. The vaccine activated skin Langerhans cells, induced Tr1 cells, and decreased myelin‐specific T cell responses. Moreover, a phase III clinical trial tested the efficacy of IV administered MBP_83-99_ in patients with secondary progressive MS. The therapy was safe but lacked therapeutic efficacy (NCT00468611) (104). Unfortunately, several peptide-based vaccines tested in MS had adverse effects. A phase II clinical trial, testing SC administration of altered peptide ligands of MBP_83-99_, resulted in systemic hypersensitivity reactions in 9% of patients (105). Additionally, in a separate phase II clinical trial, the altered peptide ligand vaccine, exacerbated MS and resulted in a 3-fold increase of CNS lesions, expansion of Th1 pathogenic MBP_83-99_-specific Tcons, and increased intramolecular epitope spreading within MBP (106). Both clinical trials were terminated due to safety concerns. Therefore, as peptide-based tolerogenic vaccines are advanced, every effort should be made to select vaccine platforms that support tolerogenic responses and minimize the risk of sensitization.

**Table 1:** Clinical trials.

Vaccine Strategy	Autoimmune Disease	Developmental Stage	Vaccine Route	Treatment Regiment	Outcomes	Ref.
Peptide vaccine consisting of encapsulated bovine myelin	Relapsing-Remitting MS	Phase I and III	Oral	Capsule taken daily for 1 year	Therapy was safe and tolerable and resulted in reduced frequency of T cells reactive with MBP. Clinically, there was a tendency for the treated group to have fewer exacerbations however a larger phase III clinical trial did not show statistically significant benefit in MS.	(99)
Peptide vaccine “ATX-MS-1467” containing MBP_30-44_, MBP_131-145,_ MBP_140-154_, MBP_83-99_	Relapsing-Remitting and Secondary Progressive MS	Phase I and II	ID or SC	Dosed every 14 days for 16 weeks or biweekly for 16 weeks	Therapy was safe and tolerable and resulted in a significant decrease in new/persisting lesions by MRI.	(100, 101)
Peptide vaccine containing MBP_85-99_, MOG_35-55_, and PLP_139-155_	Relapsing-Remitting MS	Phase I/II	Skin patch TD	Skin patch replaced once per week for 4 weeks and then once per month for 11 months	Therapy was safe and tolerable leading to a significant reduction in both MRI activity and clinical outcomes. The therapy activated dendritic Langerhans cells in the skin, induced Tr1 cells and suppressed myelin‐reactive T cell responses.	(102, 103)
Peptide vaccine “MBP8298” containing MBP_82-98_	Secondary Progressive MS	Phase III	IV	Dosed once every 6 months for 2 years	Therapy was safe but did not provide a clinical benefit compared to placebo.	(104) NCT00468611
Peptide vaccine “NBI-5788” containing altered peptide ligands derived from MBP_83–99_	Relapsing-Remitting MS	Phase II	SC	Dosed weekly for 4 months	Therapy was not safe and was discontinued due to systemic hypersensitivity reaction in 9% of the patients. Therapy resulted in the activation of Th2 responses.	(105)
Peptide vaccine “CGP77116” containing altered peptide ligands of MBP_83-99_	Relapsing-Remitting MS	Phase II	SC	Dosed weekly for 9 months	Therapy was not safe or tolerable and exacerbated MS resulting in a 3-fold increase in contrast-enhancing lesions, expansion of MBP_83-99_-specific effector T cell as well as increased intramolecular epitope spreading to disparate epitopes of MBP.	(106)
Peptide vaccine consisting of autologous PBMCs chemically coupled with MOG_1–20_, MOG_35–55_, MBP_13–32_, MBP_83–99_, MBP_111–129_, MBP_146–170_, and PLP_139–154_	Relapsing-Remitting and Secondary Progressive MS	Phase I	IV	Single dose	Therapy was safe and tolerable resulting in decreased myelin-specific T cell responses.	(107)
Nanoparticle vaccine “Xemys” consisting of CD206-targeted liposomal NP encapsulating MBP_46–62_, MBP_124–139_ and MBP_147–170_	Relapsing-Remitting and Secondary Progressive MS	Phase I	SC	Weekly injection for 6 weeks	Therapy was safe and tolerable. However, there was an increase of active gadolinium-enhancing lesions on weeks 7 and 10 in comparison with baseline.	(108)
DNA vaccine “BHT-3009” encoded the CMV promoter and full-length MBP	Relapsing-Remitting and Secondary Progressive MS	Phase I/II and II	IM	Dosed on weeks 1, 3, 5 and 9 with or without atorvastatin	Therapy was safe and tolerable leading to decreased proliferation of myelin-reactive CD4^+^ T cells from peripheral blood and a reduction in myelin-specific autoantibodies from cerebral spinal fluid. Reduced inflammatory lesions. No beneficial effects of atorvastatin.	(109) NCT00103974 and NCT00382629
DC vaccine consisting of dexamethasone treated DCs loaded with MBP_13–32_, MBP_83–99_, MBP_11–129_, MBP_146–170_, MOG_1–20_, MOG_35–55_, PLP_139–154_	Primary Progressive and Secondary Progressive MS	Phase I	IV	Three doses administered every 2 weeks	Therapy was safe and tolerable leading to the induction of Tr1 cells.	(110) NCT02283671
DC vaccine consisting of Vitamin-D3 treated DCs loaded with myelin peptides	Primary Progressive and Relapsing-Remitting MS	Phase I	Intranodal	Six doses, first 4 doses administered every 2 weeks with remaining 2 doses administered every 4 weeks	Ongoing	(111) NCT02903537
DC vaccine consisting of Vitamin-D3 treated DCs loaded with MBP_13-32_, MBP_111-129_, MBP_154-170_, PLP_139-154_, MOG_1-20_, MOG_35-55_ and MBP_83-99_	Primary Progressive and Relapsing-Remitting MS	Phase I	ID	Three doses administered every 2 weeks	Ongoing	(111, 112) NCT02618902
CAR-Treg therapy “TX200-KT02” utilizing A2-CAR-Tregs	Transplant Rejection	Phase I/II	IV	Single Dose	Ongoing	2019-001730-34
CAR-T cell therapy “Descartes-08” utilizing BCMA-CAR-T cells	Generalized Myasthenia Gravis	Phase I/II	IV	unspecified	Ongoing	NCT04146051
CAR-T cell therapy “DSG3-CAART” utilizing DSG3-CAAR T cells	Pemphigus Vulgaris	Phase I	IV	Single or multiple doses over 3 moths	Ongoing	NCT04422912
CAR-T cell Therapy utilizing CD19-CAR T cells	Systemic Lupus Erythematosus	Phase I	IV	Single Dose	Ongoing	NCT03030976

A common problem with naked peptides is they are rapidly cleared and thus exert transient effects (114). Consequently, peptide vaccines are typically administered repeatedly and often require high doses (90, 115, 116). Therefore, protein carriers are being investigated to increase the stability, half-life, and bioavailability of autoantigen peptides to increase the efficacy of peptide-based tolerogenic vaccines. Protein carriers such as mAb, cytokines, cells, and pathogen derived immunosuppressive or adhesion proteins have served as targeting moieties to introduce tethered peptides into specific immunological niches or as tolerogenic adjuvants to favor tolerance. Here we discuss tolerogenic peptide-carrier vaccine targeting and tolerogenic adjuvant strategies that generated tolerance in EAE (outlined in **Table 2**).

**Table 2:** Protein- and peptide-based tolerogenic vaccines.

Peptide-Based Tolerogenic Vaccines	Prophylactic Treatment			Therapeutic Treatment			Bystander/Infectious tolerance	Proposed Mechanism of Action	Ref.
	Route	Days	EAE Model	Route	Days	EAE Model			
Anti-DEC205-MOG_35-55_	IV	-7 and -3 or -14	C57BL/6 with lyophilized spinal cord or MOG_35-55_-specific T cell transfer	IV	7 and 11	C57BL/6 with Lyophilized spinal cord	Maybe	Antigen targeting to cross-presenting CD8^+^ DEC205^+^ DCs resulted in the induction and expansion of Tregs and decreased the percentage of IFN-γ^+^ Th1 and IL-17^+^ Th17 cells. IP and SC administration were less efficacious. Tolerance was dependent on steady state DCs and TGF-β.	(117, 118)
Anti-Langerin-MOG_29-59_	SC	-14	C57BL/6 with MOG_35-55_-specific T cell transfer	N/A	N/A	N/A	N/A	Antigen targeting to CD103^+^ Langerin^+^ migratory DCs resulted in the induction and expansion of MOG_35-55_-specific Tregs. Tolerance was dependent on vaccine-induced Tregs.	(118)
Anti-Treml4-MOG_29-59_	SC	-14	C57BL/6 with MOG_35-55_-specific T cell transfer	N/A	N/A	N/A	N/A	Antigen targeting to lymphoid resident CD8^+^ Treml4^+^ DCs failed to suppress EAE.	(118)
Anti-DCIR2-PLP_139-151_	IP	-10	SJL with PLP_139-151_	N/A	N/A	N/A	N/A	Antigen targeting to CD8^-^ DCIR2^+^ DCs resulted in the expansions of pre-existing Tregs and anergy/apoptosis of pathogenic T cells. Tolerance was dependent on steady state DCs. Anti-DCIR2-MOG_29-59_ failed to prevent MOG_35-55_-induced EAE in C57BL/6 mice (118).	(119)
Anti-Siglec-H-MOG_35-55_	IP	-1	C57BL/6 with MOG_35-55_	N/A	N/A	N/A	N/A	Antigen targeting to Siglec-H^+^ plasmacytoid DCs resulted in prolonged antigen presentation, reduced antigen-specific IFN-γ^+^ Th1 and IL-17^+^ Th17 cells and reduced MOG-specific antibodies. No increase in Tregs was observed.	(120)
Anti-BST2-MOG_35-55_	IP	-1	C57BL/6 with MOG_35-55_	N/A	N/A	N/A	N/A	Antigen targeting to BST2^+^ plasmacytoid DCs resulted in short term antigen presentation and did not suppress EAE.	(120)
GM-CSF- MBP_69-87_, PLP_139-151_, or MOG_35-55_	SC	-21, -14, and -7	Lewis Rat with MBP_69-87_, SJL with PLP_139-151_, and C57BL/6 with MOG_35-55_	SC	12, 15, 17 and 19 or 9, 10, 12, and 14	Lewis Rat with MBP_69-87_ and C57BL/6 with MOG_35-55_	N/A	Antigen targeting to DCs resulted in the induction of MOG_35-55_-specific Tregs. GM-CSF-MOG and GM-CSF-PLP_139-151_ vaccines were efficacious tolerogens when included in CFA. Tolerance was dependent on vaccine-induced Tregs and low efficiency antigen recognition.	(121, 122)
Anti-DC-ASGPR-MOG_1-125_	N/A	N/A	N/A	SC	7, 14, 21, 35 and 63	Cynomolgus macaques with Full-length human MOG	N/A	Antigen targeting to DC-ASGPR^+^ skin macrophages resulted in the induction of MOG_35-55_-sepcific Tregs, increased serum levels of TGF-β1/2 and reduced percentages of activated CD4^+^ T cells. No reduction of MOG_35-55_ specific antibodies was observed.	(123)
M-CSF-MBP_69-87_	SC	-21, -14 and -7	Lewis Rat with MBP_69-87_	SC	9, 10, 12, and 14 or 10, 11, and 13	Lewis Rat with MBP_69-87_	N/A	Antigen targeting to macrophages.	(121)
IL-2-MBP_69-87_	SC	-60, -42, and -20 or -35, -21, and -7	Lewis Rat with MBP_69-87_	SC	5, 7, and 9 or 5, 7, 9, and 11	Lewis Rat with MBP_69-87_	N/A	Antigen targeting to T cell APCs.	(124)
IL-4-MBP_69-87_	SC	-42, -28, and -14	Lewis Rat with MBP_69-87_	SC	5, 7, and 9 or 5, 7, 9, and 11	Lewis Rat with MBP_69-87_	N/A	Antigen targeting to B cells did not suppress EAE.	(124)
IFN-β-Neuroantigen or IFN-β + Neuroantigen with peptides MBP_69-87_, PLP_139-151_, or MOG_35-55_	SC	-21, -14 and -7	Lewis Rat with MBP_69-87_, SJL with PLP_139-151_, and C57BL/6 with MOG_35-55_	SC	9, 10, and 12 or 9, 10, 12, and 14 or 13, 15, 17, and 19 or 15 alone	Lewis Rat with MBP_69-87_ and C57BL/6 with MOG_35-55_	Yes	Myelin antigen presentation in IFN-β conditioned environments resulted in the induction of Tregs. Tolerance was dependent on vaccine-induced Tregs. INF-β + OVA_323-339_ in Alum ameliorate EAE induced with OVA_323-339_/MOG_35-55_ in CFA.	(125, 126)
IL-16-MBP_68-88_	SC	-31, -17 and -7 or -21, -14 and -7	Lewis Rat with MBP_69-87_	IV followed by IP	8 and 12 or 10 and 12	Lewis Rat with MBP_69-87_	N/A	Myelin antigen presentation in IL-16 conditioned environments.	(127)
24-mer S-antigen peptide-MOG_38-51_ or PLP_139-151_	IV	-7	SJL with PLP_139-151_ and C57BL/6 with MOG_35-55_	IV	7	SJL with PLP_139-151_ and C57BL/6 with MOG_35-55_	N/A	Myelin antigen presentation with immunosuppressive repeating peptides of S-antigen from *Plasmodium falciparum* increased expansion of antigen-specific Tregs. Tolerance was dependent on vaccine-induced IL-10.	(128)
pσ1-PLP_139-151_ or MOG_29-146_	Nasal	-21, -14, and -7	SJL with PLP_139-151_	Nasal	6 or 11 or 18	SJL with PLP_139-151_ and C57BL/6 with MOG_35-55_	N/A	Antigen targeting to M cells resulted in the induction of IL-10 producing Tr1, induction of IL-4 producing Tregs and decrement of IL-21, IL-17 and IFN-γ production. Tolerance was dependent on vaccine-induced Tregs and IL-4.	(129, 130)
RBC-MOG_35-55_	IV	-7	C57BL/6 with MOG_35-55_	IV	5 or 11	C57BL/6 with MOG_35-55_	N/A	Targeting of antigen to RBC recycling pathway led to pathogenic effector cell depletion and reduced the percentage of IFN-γ and IL-17 producing T cells.	(131)
Splenocytes chemically coupled with full-length MPB, MBP_72-89_, MBP_84-104_, MOG_35-55_, spinal cord homogenate, or peptide cocktail	IV	-7	SJL with PLP139-151 or spinal cord homogenate, C57BL/6 with MOG_35-55_, and Lewis Rats with MBP_72-89_	IV	3 or 4 or 16 or 30	SJL with PLP_139-151_-specific T cell transfer and SJL with peptide cocktail	N/A	Targeting of antigens to the apoptotic cell debris clearance pathway in spleen resulted in macrophage production of IL-10 and PD-L1 as well as induction of effector T cell anergy and/or deletion. Tolerance was partially dependent on vaccine-induced Tregs. IP and SC vaccination did not induce tolerance.	(132–137)

DC-targeting carrier proteins have been extensively explored in preclinical models of EAE. Myelin peptides fused to mAbs specific for DC receptors such as DEC205, DCIR2, Langerin, and Siglec-H targeted tethered myelin peptides to the corresponding receptor expressing DCs populations and prevented EAE (117–120). These antibodies were selected based on their ability to target unique DC subsets including CD8^+^ DCs, CD8^─^ DCs, CD103^+^ migratory DCs, or plasmacytoid DCs. For example, disease-specific antigen peptides fused to anti-DEC205 mAbs or single chain variable fragments (scFv) are targeted to steady state cross-presenting CD8^+^ conventional DCs and CD103^+^ migratory DCs (118), and suppressed murine models of CD4^+^ T cell-driven cartilage proteoglycan induced arthritis, DTH, T1D, and EAE as well as CD8^+^ T cell-driven contact hypersensitivity and T1D (138–142). Anti-DEC205 vaccines mediated dominant tolerance *via* the induction of antigen-specific Tregs and passive tolerance *via* the induction of autoreactive T cell anergy and apoptosis (117, 118). IV vaccination was more effective than both SC and IP. Tolerance was dependent on TGF-β (117) and immature steady state DCs (141).

Other examples of DC-targeting peptide vaccines that suppressed EAE include anti-Langerin, DCIR2 and Siglec-H fusion proteins that targeted migratory CD103^+^ DCs, CD8^-^ DCs, or plasmacytoid DCs, respectively (**Table 2**).

Carrier proteins serving as both tolerogenic adjuvants and targeting moieties have demonstrated efficacy in preclinical models of EAE. For example, the cytokine GM-CSF has been used as a tolerogenic adjuvant and a DC targeting moiety to suppress EAE. Peptides tethered to GM-CSF were specifically targeted to myeloid DCs *in vitro* (121). GM-CSF fused with either MBP_69-87_, PLP_139-151_, or MOG_35-55_ prevented EAE induced with MBP_69-87_ in Lewis rats, PLP_139-151_ in SJL mice, and MOG_35-55_ in C57BL/6 mice, respectively. These GM-CSF-neuroantigen fusion protein vaccines were effective when administered as prophylactic or therapeutic treatments in multiple rodent models of EAE (121, 143–145). GM-CSF-PLP_139-151_ or -MOG_35-55_ fusion proteins mediated tolerance in proinflammatory environments and prevented EAE when mixed directly with the encephalitogenic emulsion (144). The GM-CSF-MOG_35-55_ fusion protein increased Tregs in the blood, spleen, and lymph nodes and decreased circulating Tcon in the blood of MOG_35-55_-specific 2D2 TCR transgenic mice. Tolerance was dependent on GM-CSF-MOG_35-55_-induced Tregs (143). The mechanism by which GM-CSF-neuroantigen vaccines elicited tolerance was dependent on low efficiency TCR-antigen recognition that excluded the recruitment of the CD40-CD40L costimulatory pathway (122, 143).

Targeting myelin peptides to macrophages and DCs had tolerogenic efficacy in cynomolgus macaque and Lewis rat models of EAE. For example, anti-DC-ASGPR mAb fused with MOG_1-125_ selectively targeted MOG_1-125_ to CD163^+^ CD40^─^ resident macrophages and monocyte derived DCs (123). The anti-DC-ASGPR-MOG_1-125_ fusion protein completely protected cynomolgus macaques from MOG induced EAE when administered after sensitization but before disease onset. This vaccine decreased the percentage of activated CD4^+^ T cells, increased the percentage of MOG_35-55_-specific FOXP3^+^ Tregs, and increased levels of TGF-β1/2. Peptides can also be targeted to macrophages and DCs with the cytokine M-CSF. Recombinant fusion proteins comprised of M-CSF covalently linked with MBP_69-87_ inhibited MBP_69-87_-induced EAE in Lewis rats when administered prophylactically or therapeutically (121).

A fusion protein comprised of IL-2 and MBP_69-87_ targeted MBP_69-87_ to MHCII^+^ T cell for enhanced antigen presentation (121). The IL-2-MBP_69-87_ fusion protein prevented MBP_69-87_-induced EAE in Lewis rats when injected SC prophylactically or therapeutically (124). The tolerogenic activity was not due to the immunomodulatory properties of IL-2 because IL-2 alone did not suppress EAE. It was proposed that autoantigen presentation by MHCII^+^ CD4^+^ T cells induces T cell anergy and apoptosis (146–148). Interestingly, B cell targeting with IL-4-neurotantigen fusion protein did not elicit tolerance in EAE (121) (124).

IFN-β was used as a tolerogenic adjuvant and carrier protein because of its ability to suppresses T cell priming, inhibit Tcon proliferation, induce Tregs, and prompt tDC differentiation (149). IFN-β-neuroantigen fusion proteins were potent therapeutic or prophylactic interventions that inhibited EAE in Lewis rats and mice (125, 126, 150). The tolerogenic activity of IFN-β-neuroantigen was not due to the immunomodulatory activity of IFN-β because Lewis rats treated with an equimolar dose of IFN-β alone were not protected from EAE (150). IFN-β could also be non-covalently linked with peptides *via* hydrostatic binding in Alum to mediate tolerance. For example, a tolerogenic vaccine comprised of IFN-β + MOG_35-55_ in Alum ameliorated MOG_35-55_-induced EAE in C57BL/6 mice when administered at peak disease (125). The IFN-β + MOG_35-55_ in Alum vaccine induced Tregs in MOG_35-55_-specific 2D2 TCR transgenic mice. In addition, vaccine-induced tolerance was dependent on vaccine-induced Tregs. Interestingly, the vaccine mediated bystander suppression because IFN-β + ovalbumin (OVA_323-339_) in Alum protected mice from EAE when mice were challenged with OVA_323-339_ + MOG_35-55_ in CFA but not MOG_35-55_ in CFA. These results suggested that the IFN-β + OVA_323-339_ in Alum induced OVA-specific Treg and blocked the priming of encephalitogenic MOG_35-55_-specific T cells. Likewise, fusion proteins comprised of the immunomodulatory cytokine IL-16 and MBP_69-88_ ameliorated MPB_69-88_-induced EAE in Lewis rats when administered prophylactically or therapeutically (127). Other tolerogenic vaccine strategies included fusing myelin peptides to the immunosuppressive S-antigen from *Plasmodium falciparum* (128) or targeting myelin peptides with the viral adhesion protein pσ1, to M cells, which mediate mucosal antigen sampling and mucosal tolerance, to suppress EAE (**Table 2**) (129, 130).

Cell have been used as peptide carriers and targeting moieties. Red blood cells (RBCs) have been employed to introduce autoantigens into the RBC recycling pathway to mediate tolerance. Autoantigen loaded RBCs suppressed mouse models of EAE and T1D (131, 151). For example, RBCs carrying MOG_35-55_ protected C57BL/6 mice from MOG_35-55_-induced EAE when administered IV before or during disease onset (131). The RBC-antigen vaccine depleted antigen-specific immunocytes. Likewise, apoptotic leukocytes have been exploited to introduce autoantigens into the apoptotic cell debris clearance pathway and induced tolerance in rodent models of DTH, Theiler’s murine encephalomyelitis, allergy, experimental autoimmune thyroiditis, uveitis, neuritis, T1D, and EAE (132, 133, 152–161). For example, prophylactic IV vaccination of SJL mice with splenocytes coupled to PLP_139-151_ suppressed EAE induced with mouse spinal cord homogenate. The route of vaccine administration was critical since IV, but neither SC or IP vaccination, prevented EAE (134). The vaccine decreased antigen-specific T cell proliferation and increased the production of IL-10 and TGF-β (133). Tregs transferred tolerance from vaccinated donor mice to recipient mice and prevented the subsequent induction of EAE. Interestingly, Tregs were not required for tolerance but instead maintained long-term tolerance since Treg depletion only abrogated tolerance during late stage disease (day 63) but not during early disease (day 35). Mechanistically, it was determined that the vaccine was targeted to marginal zone macrophages in the spleen, that upregulated IL-10 and PD-L1 resulting in the expansion of Tregs and depletion of antigen-specific effectors cells (135, 162).

The success of antigen-coupled cells in preclinical models of EAE led to a phase I clinical trial testing the safety of myelin peptide-coupled PBMCs in patients with relapsing-remitting or secondary progressive MS (**Table 1**) (107). The study concluded that myelin peptide-coupled PBMC vaccination was safe and associated with decreased myelin reactive T cell recall responses in treated patients.

#### Particle-Based Vaccines

Particulate vaccines, such as nanoparticles (NP) and microparticles (MP) are being explored as protein/peptide carriers to extend autoantigen half-life and target autoantigens to distinct immunological niches. Particle-based vaccines can coordinate the delivery of tolerogenic adjuvants and autoantigens to different cell types or anatomical locations by modulating the particles size, charge, composition, and route of administration. For example, SC administered particles that are 1-6 nm, 9-100 nm, and 100 nm or greater drain to the blood, lymphatics, or form a local depot, respectively. When administered IV, particles that are 20–100 nm, 100-200 nm, and 200 nm or greater accumulate in the liver, liver/spleen or spleen, respectively (163). Ideally, particles should be biodegradable to prevent bioaccumulation and cytotoxicity (163). Here we discuss particle-based tolerogenic vaccine-targeting strategies that generate immune tolerance in rodent models of EAE (outlined in **Table 3**).

**Table 3:** Particle-based tolerogenic vaccines.

Nano and Microparticle Tolerogenic Vaccines	Prophylactic Treatment			Therapeutic Treatment			Bystander/Infectious Tolerance	Proposed Mechanism of Action	Ref
	Route	Days	EAE Model	Route	Days	EAE Model			
400-500 nm PS or PLG NP bearing PLP_139-151_	IV	-7	SJL with PLP_139-151_	IV	11 or 18	SJL with PLP_139-151_	Yes	Antigen targeting to marginal zone macrophages in the spleen led to effector T cell apoptosis and anergy. Tolerance was dependent on vaccine induced Tregs and IL-10. SC vaccination was ineffective. NP bearing PLP_139-151_ mediated infectious tolerance to PLP_178-191_-induced EAE in SJL mice.	(164)
170-220 nm PLGA NP encapsulating PLP_139-191_ and rapamycin	IV	-21 and -14	Hemophilia A mice with PLP_139-151_	IV or SC	13 or 14	SJL with PLP_139-191_	N/A	Antigen targeting to marginal zone macrophages in the spleen and myeloid APCs in the lymph nodes with rapamycin to modulate local immune environment, inhibited antigen-specific T cell proliferation and induced antigen-specific tetramer^+^ Tregs in spleen of vaccinated mice. Tregs from vaccinated mice transferred tolerance to naïve recipient mice.	(165, 166)
700-900 nm PLG NP encapsulating PLP1_39-151_ with tethered TGF-β	IV or SC	-7	SJL with PLP_139-151_	IV	13	SJL with PLP_139-151_	N/A	Antigen delivery with TGF-β to modulate local immune environment resulted in decreased DC production of IL-6 and IL-12 and increased the number of polyclonal Tregs in the liver of mice.	(167)
3.5-4.5 µm PLGA MPs loaded with MOG_35-55_ and rapamycin	NA	NA	NA	ILN	10 or 16	C57BL/6 with MOG_35-55_	N/A	Antigen delivery to lymph node with rapamycin to modulate local immune environment increased polyclonal Tregs in CNS, lymph nodes, and spleen, reduced IFN-γ and IL-17 responses and decrease lymphocyte infiltration into CNS.	(168)
20 nm Quantum dots bearing MOG_35-55_	SC	2	C57BL/6 with MOG_35-55_	NA	NA	NA	N/A	Antigen delivery to MARCO^+^ macrophages in lymph nodes increased polyclonal Tregs in the draining lymph nodes. Tolerance was dependent on NP antigen loading density.	(169)
10-20 nm Poly(maleic anhydride-alt-1-octadecene) coated iron oxide nanocrystals loaded with MOG_35-55_ or MBP_1-9_	IV	1	C57BL/6 with MOG_35-55_ or B10.PL or Tg4 with MBP_1-9_	IV	Administered at disease onset	C57BL/6 with MOG_35-55_	N/A	Antigen targeting to LSECs increased the percentage of polyclonal Tregs in the spleen. Tolerance was dependent on vaccine-induced Tregs.	(170)
60 nm Gold particles loaded with PEG, ITE, and MOG_35-55_ or PLP_139-151_	IP	-30, -29 and -28 or -16, -15 and -14 or -9, -8 and -7 or -3, -2 and -1	C57BL/6 with MOG_35-55_	IP	17, 24,31, 38 and 45 or 7 or 14 or 35	SJL with PLP_139-151_, C57BL/6 with MOG_35-55_, or NOD with MOG_35-55_	Yes	Antigen targeting to DCs with ITE to modulate local immune environment induced tolerogenic DCs and increased polyclonal Tregs in the spleen of vaccinated mice. Tregs from vaccinated mice transferred tolerance to naïve recipient mice. NP carrying ITE and MOG_35-55_ suppressed EAE induced with PLP_139-151_ in SJL x C57BL/6 F1 mice.	(171, 172)
40-50 nm Dextran coated or pegylated iron oxide NPs coated with MHCII loaded with MOG_38-49_, MOG_97-108_ or PLP_175-192_	N/A	N/A	N/A	IV or SC	14 or 21 and weekly	C57BL/6 with MOG_35-55_ or C57BL/6 IAb^null^ HLA-DR4-IE Tg mice with PLP_175–192_	Yes	Antigen presenting NPs induced Tr1 cells from memory experienced populations. NP carrying MOG_97-108_ suppressed PLP_175-192_ induced EAE. Likewise, NP bearing ubiquitous PDC or CYP2D6 peptide-MHCII complexes suppressed EAE.	(173, 174)
180-260 nm PLGA NP bearing MOG_40–54_/H-2Db-Ig dimer, MOG_35–55_/I-Ab multimer, anti-Fas, PD-L1-Fc and CD47-Fc	NA	NA	NA	IV	8, 18, 28 and 38	C57BL/6 MOG_35-55_	N/A	Antigen presenting NP with immunoregulatory molecules to modulate the local immune environment decreased MOG_35–55_-reactive Th1 and Th17 cells, increased regulatory T cells, inhibited T cell proliferation and elevated T cell apoptosis in spleen.	(175)
60-90 nm Mannosylated liposomes loaded with MBP_46–62_, MBP_124–139_, or MBP_147–170_ or combination	N/A	N/A	N/A	SC	7, 8, 9, 10, 11 and 12	Dark Agouti Rats MBP_63-81_	Yes	Antigen targeting to CD206^+^ DCs resulted in decrease anti-MBP autoantibodies and down-regulate Th1 cytokine profile in the CNS. NP carrying MBP_46–62_, MBP_124–139_, or MBP_147–170_ prevented MBP_63-81_ induced EAE in Dark Agouti rats.	(176)

One strategy is to target autoantigens to the spleen using microparticle-based (MP) tolerogenic vaccines. MP vaccines composed of either non-biodegradable polystyrene and biodegradable PLGA (400-500 nm) mimicked apoptotic cell debris and target tethered autoantigens to MARCO^+^ marginal zone macrophages in the spleen when injected IV. The MP coupled with disease-specific antigens suppressed preclinical rodent models of EAE, T1D, celiac, and allergic airway disease (164, 177–180). For example, PLP_139-151_-MP suppressed PLP_139-151_-induced EAE in SJL mice when administered prophylactically or therapeutically. The particle size was crucial because smaller (100 nm) and larger (1.75 and 4.5 µm) particles were less effective (164). Likewise, the administration route was critical because IV vaccination demonstrated robust tolerogenic activity, while IP and SC vaccination had moderate to no tolerogenic activity, respectively (178). Collectively, these data suggested that MP must be a size that is conducive for spleen targeting and marginal zone macrophage phagocytosis to elicit robust tolerance. Mechanistically, the MP induced tolerance through the induction of T cell anergy and deletion. The PLP_139-151_-MP reduced the PLP_139-151_-specific T cell recall response and decreased the percentage of PLP_139-151_-specific IFN-γ and IL-17 producing T cells *in vivo*. In addition, MP-induced tolerance was partially dependent on Tregs and IL-10 (164). Moreover, tolerogenic adjuvants, including TGF-β and rapamycin, have been added to reinforce anti-inflammatory pathways and enhance the efficacy of spleen-targeted NP/MP vaccines (165–167).

A second strategy is to target particles to the lymph nodes, a site of T cell priming. Lymph node targeting has been achieved *via* SC injection of small NP (10-100 nm) that drain to local lymph nodes and are subsequently retained (181). Additionally, intra-lymph node injection of large MPs that are too large to subsequently migrate to other anatomical sites can be used to influence the lymph node environment. For example, intra-lymph node injection of MP (3.5-4.5 µm) vaccines comprised of biodegradable PLGA bearing MOG_35-55_ and the tolerogenic adjuvant rapamycin suppressed MOG_35-55_-induced EAE in C57BL/6 mice when administered prophylactically or therapeutically. The MP were not inherently immunosuppressive, because MP bearing an irrelevant antigen and rapamycin did not suppress EAE. Tolerance was dependent on lymph node targeting because IM vaccination did not restrain EAE. The MP increased the number of Tregs in the lymph nodes and spleen of vaccinated mice (168). Moreover, a tolerogenic NP vaccine comprised of 20 nm quantum dots fused with MOG_35-55_ was targeted to MARCO^+^ macrophages in the draining lymph nodes following SC vaccination. The quantum dot-MOG_35-55_ NP vaccine prevented MOG_35-55_-induced EAE in C57BL/6 mice when administered after the encephalitogenic challenge but before disease onset. Tolerance was dependent on antigen density because NP loaded with increasing quantities of MOG_35-55_ were less efficacious. Tolerance was associated with increased numbers of Tregs in the draining lymph nodes of vaccinated mice (169).

A third strategy includes targeting tolerogenic NP to the liver. Tolerogenic NP comprised of autoantigens tethered to Poly (maleic anhydride-alt-1-octadecene) coated superparamagnetic iron oxide nanocrystals (10-20 nm) were specifically enriched in the liver and internalized by LSECs following IV administration (170). When loaded with MOG_35-55_, the LSEC targeted NP suppressed MOG_35-55_-induced EAE in C57BL/6 mice when administered IV before or during active disease (170). The LSEC-targeted NP increased the percentage of polyclonal Tregs in the spleens of vaccinated mice and Treg depletion abrogated vaccine-induced tolerance.

A fourth strategy is to target NP to DCs with tolerogenic adjuvants that promote the differentiation of tDC. AHR ligand 2-(1’H-indole-3’-carbonyl)-thiazole-4-carboxylic acid methyl ester (ITE) is an adjuvant that activates the aryl hydrocarbon receptor and promoted the differentiation of tDCs. DC targeted gold nanoparticles (60 nm) coated with disease-specific autoantigen, ITE, and PEG suppressed rodent models of EAE and T1D (171, 172, 182). The ITE-antigen-NP induced the differentiation of tDCs *in vitro*, that expressed low levels of the co-stimulatory molecules (MHCII, CD40, and CD86) and proinflammatory cytokines (IL-6 and IL-12) and increased the production of anti-inflammatory cytokines (TGF-β and IL-10) (171, 182). When administered SC or IV, the NP were selectively targeted to CD11c^+^ DCs. SC administration of the NP increased the percentage of MOG_35-55_-specific (tetramer^+^) FOXP3^+^ Tregs and IL-10 producing Tr1 cells in addition to reducing the percentage of MOG_35-55_-specific (tetramer^+^) IL-17 and IFN-γ producing Tcons in the spleen (182). The NP loaded with ITE and MOG_35-55_ suppressed MOG-induced EAE when administered IP, IV, or SC and were effective when administered prophylactically or therapeutically (171, 182). Tregs transferred tolerance from vaccinated donor mice to recipient mice and prevented the subsequent induction of EAE (171). The NP vaccine elicited bystander suppression because NP containing ITE and MOG_35-55_ suppressed PLP_139-151_-induced EAE in C57BL/6 x SJL F1 mice (182).

A fifth strategy is to directly target autoreactive T cells with artificial APC NP decorated with autoantigen-MHCII complexes. This strategy is based on the premise that chronic autoantigen stimulation in the absence of co-stimulation results in T cell anergy and expansion of suppressive Tr1 cells. NP (40-50 nm) loaded with diseases-specific autoantigen-MHCII complexes suppressed autoimmune disease in rodent models of collagen induced arthritis, autoimmune hepatitis, T1D, and EAE (173, 174, 183, 184). For example, NP bearing MOG_38-49_-MHCII complexes inhibited MOG_35-55_ induced EAE in C57BL/6 mice when administered at the peak of the disease (174). The disease resolution was associated with the conversion of antigen-experienced autoreactive T cell into suppressive Tr1 cells that expressed ICOS, LAP, CD49b, and LAG-3 as well as the anti-inflammatory cytokines IL-10 and IL-21. Effects of this vaccine were independent of FOXP3^+^ Tregs. Impressively, the autoantigen-MHCII nanoparticles elicited bystander suppression. Specifically, NP loaded with CNS-specific MOG_97-108_-MHCII but not joint-specific collagen type II (CII_259–273_)-MHCII ameliorated PLP-induced CNS autoimmunity in MHCII humanized C57BL/6 mice. Particulate size was critical because vaccines comprised of larger MP loaded with MHCII-autoantigen or smaller monomeric MHCII-autoantigen were unable to restrain EAE (174).

NP displaying peptide-MHC complexes, in which peptides are from ubiquitous autoantigens such as mitochondrial pyruvate dehydrogenase (PDC) or cytochrome P450 (CYP2D6), could induce autoantigen-specific Tr1 cells that ameliorate hepatic autoimmunity, T1D, and EAE in mice (173, 184). The consensus was that the ubiquitous self-proteins PDC and CYP2D6 are released during inflammation leading to antigen-specific CD4^+^ T cell responses. Following vaccination, the PDC- or CYP2D6-specific effector CD4^+^ T cell were converted to Tr1 cells that trafficked to sites of inflammation where PDC and CYP2D6 were released during tissue damage to mediate tolerance. To our knowledge this is the first account in which tolerance to ubiquitous autoantigens elicited organ-specific tolerance. Impressively, immunity toward vaccinia virus, influenzas and *Listeria Monocytogenes* or allogeneic colon carcinoma (CT26) and melanoma (B16/F10) liver metastases was maintained, thus the NP were not overtly immunosuppressive (173).

Tolerogenic adjuvants have been added to NP-based artificial APC platforms to enhance vaccine efficacy. For example, biodegradable PLGA nanoparticles (180-260 nm) containing surface tethered peptide-MHC complexes (MOG_40–54_/H-2D^b^-Ig dimer, MOG_35–55_/I-A^b^ multimer), regulatory molecules (anti-Fas, PD-L1-Fc), self-marker (CD47-Fc), and encapsulated TGF-β1 modulated T cell responses to induce tolerance. These NP suppressed MOG_35-55_-induced EAE in C57BL/6 mice when administered during disease onset. Tolerogenic NP without neuroantigen but with regulatory molecules lacked therapeutic activity in EAE suggesting the NP were not inherently immunosuppressive. Mechanistically, the MOG-MHC loaded NP decreased the percentage of MOG_35–55_-reactive Th1 and Th17 cells and increased the percentage of Tregs in the spleen (175).

One tolerogenic NP vaccine has been advanced into a clinical trial for MS (108). The NP was composed of mannosylated liposome unilamellar vesicles that were 60-90 nm in diameter and encapsulated MBP peptides. Preclinical studies determined that mannosylated liposome NP were selectively phagocytosed by DCs *in vitro*, *via* the mannose receptor CD206. The mannosylated liposome NP loaded with either MBP_46–62_, MBP_124–139_, or MBP_147–170_ as well as a mixture of the three peptides together prevented MBP_63-81_-induced EAE in Dark Agouti rats when administered SC at disease onset (176). These NP decreased serum anti-MBP autoantibodies and down-regulated Th1 cytokine profile. Following the preclinical success of mannosylated liposome NP, a phase I clinical trial was initiated in patients with relapsing-remitting or secondary progressive MS. Patients were treated SC with ascending doses of “Xemys” a mannosylated liposome NP loaded with MBP_46–62_, MBP_124–139_, and MBP_147–170_ (**Table 1**). The study determined that Xemys was safe and well tolerated. However, there was an increase of active CNS lesions on weeks 7 and 10 in comparison with baseline.

#### DNA-Based Vaccines

DNA-based vaccines are made of DNA vectors that include nucleic acid sequences encoding target antigens. When injected, the DNA-based vaccine is internalized by local or target cells and translated to generate *in situ* protein products that are subjected to traditional antigen presentation on MCHII. Presentation of DNA vaccine protein products (e.g. peptide) on MCHII then elicits immunogenic or tolerogenic immune responses. These DNA-based vaccines have been administered as naked plasmid DNA or as DNA constructs packaged in cationic lipids, liposomes, microparticles, and viruses to mediate DNA uptake and ectopic expression of the encoded autoantigens (185). Numerous DNA-based tolerogenic vaccines have been devised that induce antigen-specific tolerance in animal models of autoinflammatory disease (186), including murine models of EAE (185). However, several DNA-based vaccines encoding myelin antigens failed to induce tolerance and instead resulted in sensitization and exacerbated EAE (187–189). Therefore, several strategies have been conceived to specifically tailor DNA-vaccines to promote immune tolerance and prevent sensitization. These strategies include: (1) targeting DNA vaccines to anatomical sites that favor immune regulation such as the skin, muscle, and liver; (2) limiting expression of DNA vaccine to DC subsets; (3) co-expressing or administering tolerogenic adjuvants alongside DNA vaccines; and (4) modifying DNA vaccines to reduce the number of immunogenic CpG motifs to inhibit proinflammatory TLR-9 activation. Here we discuss DNA-based tolerogenic vaccine strategies that generate immune tolerance in rodent models of EAE (outlined in **Table 4**).

**Table 4:** DNA-based tolerogenic vaccines.

DNA/RNA-Based Tolerogenic Vaccines	Prophylactic Treatment			Therapeutic Treatment			Bystander/Infectious Tolerance	Proposed Mechanism of Action	Ref.
	Route	Days	EAE Model	Route	Days	EAE Model			
DNA pCMV full-length MOG or full-length MBP	IM	-28 and -14	C57BL/6 with MOG_35-55_	IM	10, 24 and 32	C57BL/6 with MOG_35-55_	Yes	Ectopic expression of MOG_35-55_ in muscle resulted in decreased Th1/Th2 responses, increased FOXP3 mRNA in CNS and increased Tregs in spleen. The DNA vaccine encoding full-length MBP suppressed MOG_35-55_-induced EAE.	(190)
DNA pfascin1 MOG_35-55_ coadministered with DNA pCMV IL-10	ID	-21, -14, and -7	C57BL/6 with MOG_35-55_	N/A	N/A	N/A	N/A	Ectopic expression of MOG_35-55_ in skin resident DCs with IL-10 to modulate the local immune environment reduced IFN-γ and IL-17 producing T cell infiltration in CNS. No detectable increase in Tregs.	(191)
DNA liver-specific promoter full-length MOG packaged in AAV coadministered with or without rapamycin	IV	-200 or -14	C57BL/6 with MOG_35-55_	IV	Treatment administered at Score 1.4, 3.0, or 3.5	C57BL/6 with MOG_35-55_	N/A	Ectopic expression of MOG in liver resulted in increased percentages of MOG_35-55_ tetramer^+^ Tregs in spleen, increased polyclonal Tregs in blood and reduced autoantibody production.	(192)
DNA pCMV MOG_35-55_ coadministered with FK506	IM	-7	C57BL/6 with MOG_35-55_	IM	3, 7, 17 and 20	C57BL/6 with MOG_35-55_	N/A	Ectopic expression of MOG_35-55_ and coadministration of FK506 to modulate immune environment resulted in increased Tregs in spleen and spinal cord of vaccinated mice.	(193, 194)
DNA pCMV MBP_68-86_ coadministered DNA pCMV IL-10	N/A	N/A	N/A	IM	10 and/or 12	Lewis Rat with MBP_68-86_	Yes	Ectopic expression of MBP_68-86_ with IL-10 to modulate local immune environment led to the induction of Tr1 cells in lymph nodes. The MBP_68-86_-DNA with IL-10-DNA vaccine suppressed MBP_87-99_-induced EAE.	(195)
DNA pCMV MOG_35-55_ coadministered with DNA pCMV TGF-β	ID	-21, -14, and -7	C57BL/6 with MOG_35-55_	N/A	N/A	N/A	N/A	Ectopic expression of MOG_35-55_ in skin with TGF-β to modulate the local immune environment resulted in reduced IFN-γ and IL-17 producing T cell infiltration into the CNS. No detectable increase in Tregs.	(191)
DNA pCMV PLP_139-151_ or full-length MOG coadministered with DNA pCMV IL-4	IM	-10 and -17	SJL with PLP_139-151_	IM	14 and 21 or 18 and 27	C57BL/6 with MOG_35-55_	N/A	Ectopic expression of PLP_139-151_ with IL-4 to modulate local immune environment reduced the proliferative response of PLP_139-151_-specific T cells, increased IL-10 and IL-4 and decreased IFN-γ production. Tolerance was transferred from donor to recipient mice via CD4^+^ (Th2) T cells.	(196)
RNA encoding MOG_35-55_ or PLP_139-151_ loaded into DC-targeted liposomes (200-400 nm)	N/A	N/A	N/A	N/A	7 and 10 or when mice reached EAE score of 1-2	C57BL/6 with MOG_35-55_ or C57BL/6 x SJL F1 with PLP_139-151_ or peptide cocktail	Yes	Ectopic expression of MOG_35-55_ in DCs decreased the percentage of IFN-γ^+^ Th1, IL-17^+^ Th17 T cells, decreased the percentage of MOG_35-55_-specific CD4^+^ T cells in the CNS and increased the percentage of MOG_35-55_-specific Tregs in the spleen of vaccinated mice. The MOG_35-55_-RNA vaccine prevented PLP_139-151_ and PLP_139-151_ or peptide cocktail-induced EAE in C57BL/6 x SJL F1 mice.	(197)

The anatomical site of autoantigen recognition can influence tolerance. Therefore, DNA-based vaccines are often introduced into anatomical sites that are immunologically quiescent (e.g. muscle) or promote Treg responses (e.g. skin and liver). For example, intramuscular (IM) vaccination of C57BL/6 mice with a DNA-vaccine encoding full-length MOG under the ubiquitous CMV promoter suppressed MOG_35-55_-induced EAE when administered prophylactically or therapeutically (197). The vaccine increased the percentage of polyclonal Tregs in the spleen and increased the expression of the Treg-specific transcription factor FOXP3 in the CNS. Splenocytes from MOG-DNA vaccinated mice produced less INF-γ, IL-17, and IL-4 following re-stimulation with MOG_35-55_. Of note, a full-length MBP-DNA vaccine elicited bystander suppression because the MBP-DNA vaccine suppressed MOG_35-55_-induced EAE when administered before disease induction. However, a full-length PLP-DNA vaccine failed to suppress MOG_35-55_-induced EAE as a therapeutic or prophylactic vaccine (197). In addition, DNA-based vaccines targeted to skin resident DCs suppressed EAE (198) (**Table 4**).

DNA-based tolerogenic vaccines encoding autoantigens have also been targeted to the liver. For example, an adeno-associated virus (AAV) packaged DNA-vaccine encoding full-length MOG under the control of a liver specific promoter resulted in the stable and restricted expression of MOG in the liver of C57BL/6 mice (199). The AAV liver targeted MOG-DNA vaccine elicited robust and lasting (>335 days) tolerance to MOG_35-55_-induced EAE and was effective when administered as prophylactic or therapeutic vaccine. The vaccine increased the percentage of MOG_35-55_-specific tetramer^+^ Tregs in the spleen and increased the abundance of polyclonal Tregs in the blood of vaccinated mice. Additionally, DNA-based tolerogenic vaccines have been co-administered with the tolerogenic adjuvants FK506 and rapamycin to suppress EAE (199–201) (**Table 4**).

Furthermore, DNA vaccines encoding autoantigens in conjunction with the immunoregulatory cytokines IL-10, TGF-β, or IL-4 were tested. For example, co-administration of separate plasmids encoding MBP_68-86_ or IL-10 under the CMV promoter suppressed MBP_68-89_-induced EAE in Lewis rats when administered prophylactically or therapeutically (202). The vaccine was associated with the induction of Tr1 cells. Interestingly, the tolerogenic vaccine demonstrated bystander suppression because the IL-10 + MBP_68-86_-DNA vaccine blocked EAE induced with MBP_87-99_ (202). DNA-based vaccines encoding autoantigens in conjunction with DNA vaccines encoding TGF-β (198) or IL-4 (203) suppressed EAE in rodents (**Table 4**).

The success of DNA-based tolerogenic vaccines in rodent models of EAE led to the development of a phase I/II and II clinical trials testing “BHT-3009”, a DNA-based tolerogenic vaccine encoding the CMV promoter and full-length MBP (**Table 1**). The DNA backbone contained reduced numbers of immunostimulatory CpG motifs to limit TLR-9 activation. The BHT-3009 vaccine was injected IM into patients with relapsing-remitting or secondary progressive MS (NCT00103974) (109). The DNA vaccine was found to be safe and decreased the number of CNS lesions in patients, however the differences did not reach statistical significance. Interestingly, the vaccine decreased the antigen-specific T and B cell response not only to MBP, but also PLP and MOG, suggesting that DNA-based vaccination may promote bystander suppression in humans.

#### RNA-Based Vaccines

RNA-based tolerogenic vaccines have been explored (outline in **Table 4**) (204). Extracellular and double stranded RNA molecules are inherently proinflammatory and trigger TLR activation, DC maturation, IFN-α production, and Th1 responses. Therefore, efforts were made to reduce the proinflammatory nature of RNA-vaccines by replacing uridine with 1-methylpseudouridine (m1Ψ) to abrogate TLR-3, TLR-7, and TLR-8 activation (205) and by removing double-stranded RNA contaminants to abrogate TLR-7 activation (206). In addition, RNA-based vaccines have been loaded into DC targeting liposomes to specifically introduce the RNA-based vaccine into DCs for translation and MHCII presentation (207). The nanoparticle-formulated m1Ψ-modified single stranded mRNA vaccine that encoded MOG_35-55_ inhibited MOG_35-55_-induced EAE in C57BL/6 mice when administered before or after disease development (204). The vaccine decreased the percentage of IFN-γ^+^ Th1, IL-17^+^ Th17 and MOG_35-55_-specific (tetramer^+^) CD4^+^ T cells in the CNS and increased the percentage of MOG_35-55_-specific (tetramer^+^) FOXP3^+^ Tregs in the spleen. The vaccine mediated bystander suppression as the MOG_35-55_-RNA vaccine also prevented PLP_139-151_-induced EAE in C57BL/6 x SJL F1 mice when administered before disease onset. Likewise, the MOG_35-55_-RNA tolerogenic vaccine ameliorate EAE induced with a cocktail of MOG_35-55_, PLP_139-151_, PLP_178-191_, MBP_84-104_, and Myelin-associated oligodendrocyte basic protein (MOBP_15-36_) in C57BL/6 x SJL F1 mice when administered as a prophylactic vaccine. These results suggest that RNA-based tolerogenic vaccines encoding a single myelin epitope can induced antigen specific-Tregs that mediate bystander suppression toward multiple noncognate myelin epitopes to control CNS autoimmunity in mice.

#### DC-Based Vaccine: Autologous DC as a Vaccine

Transplant of autologous cells including stem cells, Tregs, Tr1, iTr35, Bregs, DC, myeloid suppressor cells, microglia, and macrophages among others, suppressed EAE (66, 208–214). Autologous autoantigen loaded DCs have garnered interest as cell-based tolerogenic vaccine strategy due to the unique capacity of DCs to favor Treg response under inflammatory conditions. DCs can be loaded with autoantigens to confers antigen-specificity and treated with pharmacological agents and biologics to derive stable immunosuppressive tDCs. Determining the best methods for generating robust tDCs are currently being investigated. In **Table 5**, we summarized DC-based tolerogenic vaccine strategies that generate immune tolerance in rodent models of EAE. Pharmacological agents that inhibit the NF-kB pathway (190), a selective JAK1/JAK3 inhibitor Tofacitinib (191), a selective JAK3/STAT5 inhibitor BD750 (192), cytotoxic agents dexamethasone and/or minocycline (193), VitD3 (215), and biologics IL-27 (195) and IL-10 (196) were used to differentiate tDCs. When loaded with autoantigens these tDCs suppressed EAE.

**Table 5:** DC-based tolerogenic vaccines.

DC-Based Tolerogenic Vaccines	Prophylactic Vaccination			Therapeutic Vaccination			Bystander/Infectious Tolerance	Proposed Mechanism of Action	Ref.
	Route	Days	EAE Model	Route	Days	EAE Model			
Andrographolide or rosiglitazone treated DCs loaded with MOG_35-55_	IV	-7 and -14	C57BL/6 with MOG_35-55_	N/A	N/A	N/A	N/A	Andrographolide or rosiglitazone treated DCs had reduced expression of CD40 and CD86. When loaded with MOG_35-55_ the DCs increased FOXP3 mRNA expression in spleen, decreased MOG-specific antibodies, and decreased MOG_35-55_ specific T cell production of INF-γ and IL-2 and suppress EAE.	(190)
Tofacitinib treated DCs loaded with MOG_35-55_	N/A	N/A	N/A	IV	7, 11 and 15	C57BL/6 with MOG_35-55_	N/A	Tofacitinib treated DCs exhibited reduced expression of CD80, CD86, CD83, CD40, IL-1, IL-6, IL-12, IL-23, and TNF-α. When loaded with MOG_35-55_ the DCs reduced the percentage of IL-17^+^ and IFN-*γ* ^+^ T cells, increased the percentage of Tregs in spleen and ameliorated EAE.	(191)
BD750 treated DCs loaded with MOG_35-55_	N/A	N/A	N/A	IV	7, 11 and 15	C57BL/6 with MOG_35-55_	N/A	BD750 treated DCs exhibited reduced expression of CD80, CD86, CD83 and CD40. When loaded with MOG_35-55_ the DCs reduced the frequency of Th1 and Th17, increased the percentage of Tregs in spleen and suppressed EAE.	(192)
Dexamethasone and/or minocycline treated DCs loaded with MOG_35-55_	IV	-7 and -3	C57BL/6 with MOG_35-55_	N/A	N/A	N/A	N/A	Dexamethasone and/or minocycline treated DCs exhibited reduced levels of MCHII, CD80, CD86, TNF-α, IL-1β, IL-6, and IL-12 and increased levels of PDL-1, ICOSL and IL-10 and induced Tregs in vitro. When loaded with MOG_35-55_ these DCs suppressed EAE.	(193)
VitD3 treated DCs loaded with MOG_35-55_	N/A	N/A	N/A	IV	10, 13 and 16	C57BL/6 with MOG_35-55_	N/A	VitD3 treated DCs exhibited reduced levels of MHCII, CD80, and CD83. When loaded with MOG_35-55_ these DCs reduced Th1 and Th17 cells infiltration into spinal cord, increased the percentage of Tregs in spleen and lymph node, increased the number of Bregs in spleen and suppressed EAE.	(194)
Il-27 treated DCs loaded with PLP_139-151_	N/A	N/A	N/A	IV	20, 24, 28 and 32	SJL with PLP_139-151_	Maybe	IL-27 treated DCs exhibited reduced levels of MHCII, CD40, CD80, CD86, IL-6, IL-12 and IL-23, increased levels of TGF-β, IL-10 and IFN-β, induced Tregs and Tr1 cells. When loaded with PLP_139-151 _ the DCs suppressed EAE and PLP_178-191_ T cell responses.	(195)
IL-10 and LPS treated DCs loaded with MOG_35-55_	IV	-21	C57BL/6 with MOG_35-55_	N/A	N/A	N/A	N/A	IL-10 and LPS treated DCs exhibited decreased IL-12 production and impaired antigen-specific T cell proliferation in vitro When loaded with MOG_35-55_ these DCs suppressed EAE.	(196)

Several DC-based tolerogenic vaccines have been advanced into clinical trials for the treatment of MS (**Table 1**). First, a phase I clinical trial tested the safety of IV administered myelin peptide loaded DCs in patients with primary or secondary progressive MS (NCT02283671). To generate DCs, PBMC monocytes were cultured with IL-4, and GM-CSF and treated with dexamethasone to induce a tDC phenotype. The tDCs were further treated with IL-1β, IL-6, TNF-α, and prostaglandin E2, and loaded with a mixture of the myelin peptides, MBP_13–32_, MBP_83–99_, MBP_11–129_, MBP_146–170_, MOG_1–20_, MOG_35–55_, and PLP_139–154_. The myelin-loaded tDC vaccine was safe because patients remained stable in terms of relapse and disability. The DC vaccination increased IL-10 production and increased Tr1 cells (110). In addition, two more phase I clinical trials are underway testing the feasibility and safety of intranodal (IN) and intradermal (ID) administration of VitD3 treated DCs loaded with a mix of myelin peptides (NCT02618902 and NCT02903537) (27, 213).

## T Cell Therapy

Recent progress in single cell sorting, cell monoculture techniques, and genetic engineering paired with the clinical success of chimeric antigen receptor (CAR)-T cell therapy in treating hematologic cancer has renewed enthusiasm for the use of T cell-based therapies to treat autoimmune diseases. Treg adoptive transfer therapy has become a major focus of cell-based therapy as these cells suppress antigen-specific autoimmunity. Although there are multiple substantial barriers to overcome, T cell therapy is an elegant concept: as these therapies would directly provide antigen-specific Tregs to suppress immune response or cytotoxic T cells to remove autoreactive immune cells, a common goal among tolerogenic vaccine platforms.

### Polyclonal Treg Cell Therapy

Mounting evidence suggest that Treg adoptive transfer may be an efficacious treatment for inflammatory autoimmune diseases. Indeed, the adoptive transfer of polyclonal Tregs suppresses numerous animal models of autoimmunity, allergic disease, and transplant rejection (216). These realizations initiated multiple clinical trials investigating autologous polyclonal Treg transfer as a treatment for autoimmune disease and transplant rejection. In these clinical trials, polyclonal Tregs were purified from the patient’s blood, expanded *ex-vivo*, and then subsequently reinfused into patient as an autologous Treg cell therapy. Polyclonal Tregs have been or currently are being tested in patients with T1D, pemphigus vulgaris (PV), autoimmune hepatitis, systemic lupus erythematosus (SLE), Crohn’s disease, and organ transplant [reviewed here (217)]. Completed clinical trials in T1D (218) and transplant rejection (219) have revealed that polyclonal Treg therapy is safe and, in some cases, demonstrated modest but limited efficacy.

### Antigen-Specific Treg Cell Therapy

Antigen-specific Tregs, suppress antigen-specific immune responses more potently compared to polyclonal Tregs, and are being investigated as cell-based therapy for autoimmunity (220, 221). However, multiple technical hurdles must be overcome for widespread use in the clinic. First, generating large quantities of antigen-specific Tregs is difficult due to their low precursor frequency *in vivo* and their limited proliferative potential. Moreover, Treg cell surface markers are limited, thus Tcon contamination poses a concern. Nevertheless, preclinical studies utilizing TCR transgenic mice as a source of antigen-specific Tregs demonstrated the potential use of antigen-specific Tregs to treat autoimmunity. For example, TCR transgenic Tregs that recognized MOG_35-55_, PLP_139-151_, and MBP_1-9_ were able to suppress EAE driven with each respective neuroantigen (58, 222, 223). These antigen-specific Tregs suppressed disease not only as a prophylactic, as did polyclonal Tregs, but also as a therapeutic treatment during active disease. For example, MBP_1-9_-specific Tregs completely inhibited MBP_1-9_-induced EAE in B10.PL while non-specific polyclonal Tregs only transiently inhibited EAE (222). Furthermore, antigen-specific Tregs exhibited bystander suppression to suppress CNS autoimmunity elicited against nonrelated myelin antigens. For example, MBP_1-9_-specific Tregs partially inhibit EAE induced with PLP_139-151_ (222). Likewise, PLP_139-151_-specific Tregs were able to restrain EAE induced with a disparate epitope of PLP_178-191_ (223). Together these studies support the development of antigen-specific Treg therapy for autoimmunity.

### Engineered Treg Cell Therapy

Expression of chimeric antigen receptors (CAR) or synthetic TCR confers antigen-specificity to polyclonal Tregs. CAR- and TCR-Tregs are being developed as a strategy to suppress pathogenic immune responses (outlined in **Table 6**).

**Table 6:** T cell therapies for the treatment of inflammatory disease.

T cell Therapeutic	Disease Model	Extracellular Domain	Trans-membrane Domain	Intracellular Domain and Payload	T Cell Subset Utilized as Therapeutic	Outcomes	Ref.
CD4^+^ A2-CAR-Tregs	Transplant Rejection	Anti-HLA-A2 scFv (Clone BB7.2)	CD28	CD28-CD3ζ	Human CD4^+^ CD45RO^low^ CD45RA^high^ CD25^high^ Tregs	5-10 x10^5^ human CD4^+^ A2-CAR-Tregs suppressed xenogeneic GVHD in NSG mice when administered with effector HLA-A2^+^ T cells.	(224)
CD4^+^ A2-CAR-Tregs	Transplant Rejection	Anti-HLA-A2 scFv (phage display library)	CD28	CD28-CD3ζ	Human CD4^+^ CD25^+^ Treg	1 x10^6^ human CD4^+^ A2-CAR-Tregs prevented human HLA-A2^+^ skin graft rejection in BRG mice when administered with HLA-A2^-^ effector T cells.	(225)
CD4^+^ A2-CAR-Tregs	Transplant Rejection	Anti-HLA-A2 scFv (phage display library)	CD8	CD28-CD3ζ	Human CD8^−^ CD4^+^ CD25^high^ CD127^low^ Tregs	1 x10^6^ human CD4^+^ A2-CAR-Tregs prevented human HLA-A2^+^ skin graft rejection in NRG mice when administered with HLA-A2^-^ effector T cells.	(226)
CD4^+^ MOG-CAR-FOXP3-T cells	Multiple Sclerosis	Anti-MOG scFv (Clone 8.18 C5)	CD3	CD3ζ-CD28 Payload FOXP3	Murine CD4^+^ T cells	1 x10^5^ murine CD4^+^ MOG-CAR-FOXP3-T cells suppressed MOG_35-55_ induced EAE in C57BL/6 mice when administered after disease onset.	(36)
CD4^+^ MOG- or MBP-CAR-Tregs	Multiple Sclerosis	Anti-MOG or MBP scFv (phage display library)	CD28	CD28-CD3ζ	Human CD4^+^ CD25^high^ and CD127^low^ Tregs	1 x10^6^ human CD4^+^ MOG- and MBP-CAR-Tregs mixed 50:50 suppressed MOG_35-55_-induced EAE in C57BL/6 mice when administered 7 days after disease induction.	(227)
CD4^+^ TNP-CAR-Tregs	Colitis	Anti-TNP (Clone Sp6)	CD28	CD28-FcRγ	Murine CD4^+^ CD25^high^ Tregs from TNP-CAR Tg mice	1 x10^6^ murine CD4^+^ TNP-CAR-Tregs suppressed TNBS induced colitis in BALB/c or C57BL/6 mice when administered 16 hours after disease induction.	(228)
CD4^+^ Insulin-CAR-FOXP3-T cells	Type 1 Diabetes	Anti-Insulin scFv (phage display library)	CD8	CD28-CD3ζ Payload FOXP3	Murine CD4^+^ T cells	2.5 x10^6^ murine CD4^+^ Insulin-CAR-FOXP3-T cells failed to prevent the onset of diabetes in prediabetic NOD mice.	(37)
CD4^+^ FVIII-CAR-Tregs	Anti-FVIII responses in Hemophilia A	Anti-FVIII scFv (phage display library)	CD28	CD28-CD3ζ	Human CD4^+^ CD25^high^ CD127^low^ CD45RA^+^ Tregs	1-2 x10^6^ human CD4^+^ FVIII-CAR-Tregs suppressed the formation of FVIII antibodies in FVIII exon 16 knockout x HLA-DRB1 mice when administered on the same day as FVIII sensitization.	(229)
CD4^+^ FVIII-CAR-T cells	Anti-FVIII responses in Hemophilia A	Anti-FVIII scFv (phage display library)	CD28	CD28-CD137-CD3ζ Payload FOXP3	Murine CD4^+^ T cells	4 x10^5^ murine CD4^+^ FVIII-CAR-FOXP3-T cells suppressed the formation of FVIII antibodies in mice FVIII exon 16 knockout mice receiving FVIII gene therapy.	(230)
CD4^+^ CEA-CAR-Tregs	Allergic Disease	Anti-CEA scFv (Clone SCA431)	CD4	CD28-CD3ζ	Murine CD4^+^ CD25^+^ Tregs from CEA-CAR Tg mice	1 x10^6^ murine CD4^+^ CEA-CAR-Tregs suppressed hyper-reactivity, mucus production, and eosinophilia in CEA Tg mice with OVA induced experimental asthma when administered 7 days after the first sensitization.	(231)
CD4^+^ UniCAR- Tregs	Tumor Rejection Suppression	Anti-E5B9 scFv (Clone 5B9) + anti-PSCA-E5B9	CD28	CD28-CD3ζ or CD137-CD3ζ	Human CD4^+^ CD25^+^ CD127^dim^ CD45RA^+^ Tregs	1 x10^6^ human CD4^+^ PSCA-UniCAR-Tregs with the CD137-CD3ζ signaling domains suppressed the rejection of PC3-PSCA tumors in NMRI^nu/nu^ mice when administered alongside PSCA-CAR-T cells.	(232)
CD4^+^ FITC-CAR-Tregs	Transplant Rejection	Anti-FITC scFv (Clone 1X9Q) + anti-H-2^d^-FITC	CD28	CD28-CD3ζ	Murine CD4**^+^** CD25^high^ GFP^+^ Tregs from FoxP3^luc/GFP^ mice	1.5 x10^6^ murine CD4^+^ anti-H-2^d^-Fitc-CAR-Tregs increased H-2^d^ islet grafts survival in C57BL/6 (H-2^b^) recipients.	(233)
CD8^+^ A2-CAR-Tregs	Transplant Rejection	Anti-HLA-A2 scFv (Clone BB7.2)	CD28	CD28-CD3ζ	Human CD8^+^ CD45RC^low^ Tregs	1.5-5 x10^6^ human CD8^+^ A2-CAR-Tregs prevented human HLA-A2^+^ skin graft rejection in NRG mice when administered with HLA-A2^-^ effector T cells.	(234)
CD4^+^ MBP_89-101_-I-As- CAAR-Tregs	Type 1 Diabetes	I-As-MBP_89-101_	I-As	CD3ζ	Murine CD4^+^ CD25^+^ Tregs from MBP_89-101_-IA^s^-ζ Tg mice	1 x10^6^ murine CD4^+^ MBP_89-101_-I-As-CAAR-Tregs suppressed MBP_89-101_ induced EAE in SJL mice when administered before or after disease onset.	(235)
CD4^+^ OVA-BAR-Tregs	Allergic Disease	Full-length OVA	CD28	CD28-CD3ζ	Murine TGF-β induced CD4^+^ Tregs or human CD4^+^ CD25^high^ CD127^low^ Tregs	5 x10^6^ murine or 1 x10^6^ human CD4^+^ OVA-BAR-Tregs protected BALB/c mice from OVA induced anaphylaxis. In addition, five million murine CD4^+^ OVA-BAR-Tregs suppressed anti-OVA IgE mast cell mediated anaphylaxis.	(236)
CD4^+^ FVIII-BAR-Tregs	Anti-FVIII responses in Hemophilia A	FVIII A2 and C2 domains	CD28	CD28-CD3ζ	Human CD4^+^ CD25^high^ CD127^low^ Tregs	1-2 x10^6^ human CD4^+^ FVII-BAR-Tregs suppressed the formation of FVIII antibodies in FVIII exon 16 knockout mice when administered before and after sensitization.	(237)
CD4^+^ OVA-TCR-Tregs or OVA-TCR-FOXP3-T cells	Rheumatoid Arthritis	OVA-specific TCR α and β	TCR α and β chains	TCR α and β chains Payload FOXP3	Murine CD4^+^ CD25^+^ Tregs	1.5 x10^6^ murine CD4^+^ OVA-TCR-Tregs or OVA-TCR-FOXP3-T cells suppressed methylated BSA induced arthritis in C57BL/6 mice following a rechallenge with methylated BSA+ OVA.	(238)
CD4^+^ MBP-TCR-Tregs	Multiple Sclerosis	MBP_85-99_-specific TCR α and β	TCR α and β chains	TCR α and β chains	Human CD4^+^ CD25^high^ CD127^low^ Tregs	2 x10^6^ human CD4^+^ MBP-TCR-Tregs suppressed MOG_35-55_ induced EAE in HLA-DR15 Tg mice when administered 7 days after disease induction.	(239)
CD4^+^ FVIII-TCR-Tregs	Anti-FVIII responses in Hemophilia A	FVIII_2191-2220_ -specific TCR α and β	TCR α and β chains	TCR α and β chains	Human CD4^+^ CD25^high^ CD127^low^ Tregs	1-2 x10^6^ human CD4^+^ FVIII-TCR-Tregs suppressed FVIII antibody formation in FVIII exon 16 knockout mice crossed to human HLA-DRB1 mice.	(240)
CD8^+^ CD19-CAR-T cells	Systemic Lupus Erythematosus	Anti-CD19 scFv (Clone 1D3)	CD28	CD28-CD3ζ	Murine CD8^+^ T cells	1 x10^6^ murine CD8^+^ CD19-CAR-T cells suppressed SLE when administered before or after the development of disease in MRL-lpr and MZB/w mice, respectively.	(241)
CD8^+^ FcϵRIα-CAR-T cells	Allergic Diseases	FcϵRIα and reduced affinity muteins	CD3ζ	CD3ζ	Human CD8^+^ T cells	Human CD8^+^ FcϵRIα-CAR-T cells killed cells expressing transmembrane IgE.	(242)
CD8^+^ Anti-InsB_9-23_-I-A^g7^-CAR-T cells	Type 1 Diabetes	Anti-InsB_9-23_-I-A^g7^ scFv (clone mAb287)	CD28	CD28-CD3ζ or CD28-CD137-CD3ζ	Murine CD8^+^ T cells	3-5 x10^6^ murine CD8^+^ Anti-InsB_9-23_-I-A^g7^-CAR-T cells suppressed the development of diabetes when transferred to prediabetic NOD mice.	(243)
CD8^+^ InsB_15-23_-β2m-CAAR-T cells	Type 1 Diabetes	InsB_15-23_-β2m	CD3ζ	CD3ζ	Murine CD8^+^ T cells	1 x10^7^ murine CD8^+^ InsB_15-23_-β2m-CAAR-T cells suppressed the development of diabetes when transferred to prediabetic NOD mice.	(244)
DSG3-CAAR-T cells	Pemphigus Vulgaris	Dsg3 extracellular domains 1-3, 1-4 or 1-5	CD8	CD137-CD3ζ	Human T cells	Human Dsg3-CAAR-T cells suppressed Dsg3-specific hybridoma driven GVHD in NSG mice.	(245)
MuSK-CAAR-T cells	Myasthenia Gravis	MuSK extracellular domain	N/A	CD137-CD3ζ	Human T cells	Human MuSK-CAAR-T cells suppressed the proliferation of MuSK-specific B cells in NSG mice.	(246)

#### CAR-Treg Cell Therapy

***CD4^+^ CAR-Tregs:*** CD4^+^ CAR-Tregs are being investigated to treat GVHD and transplant rejection. The hypothesis is that Tregs expressing a CAR that recognizes a graft-specific MHCI haplotype can suppress allogenic graft transplant rejection. Several MHCI-recognizing CAR constructs have been devised. One such CAR construct encoded an extracellular HLA-A2-specific scFv, transmembrane CD28 domain, and the intracellular signaling domains of CD28 and CD3ζ (224). Human Tregs transduced with this receptor (A2-CAR-Tregs) were activated, proliferated, and upregulated the Treg activation markers when co-cultured with HLA-A2 expressing cells. The A2-CAR-Tregs selectively interacted with HLA-A2^+^ PBMCs and suppressed allogenic Tcon responses against HLA-A2 better than polyclonal Tregs *in vitro*. The human HLA-A2-CAR-Tregs were also superior to polyclonal Tregs at preventing xenogeneic GVHD, when transferred together with HLA-A2^-^ Tcons to NSG mice (224). In two independent systems, human A2-CAR-Tregs were also able to inhibit allogenic rejection of human HLA-A2^+^ skin grafts more effectively than polyclonal Tregs in BRG or NSG mice (225, 226). In addition, an extensive panel of intracellular co-signaling domains were tested in combination with the intracellular CD3ζ signaling domain in human A2-CAR-Tregs (247) and determined that the CD28 co-signaling domain was the most potent at suppressing HLA-A2^+^ PBMC-mediated GVHD in NSG mice. These murine studies led to the initiation of a phase I/II clinical trial investigating the safety and efficacy of A2-CAR-Tregs in MHCI-mismatched HLA-A2^+^ kidney transplant patients in end-stage renal failure (**Table 1**) (EudraCT number 2019-001730-34).

CD4^+^ CAR-Tregs are also being investigated as an intervention for organ-specific autoimmune diseases in mice. The hypothesis is that CAR-Tregs can be targeted to specific-organs and suppress organ-specific autoimmunity using CARs that recognize organ-restricted autoantigens. For example, CAR-Tregs that recognized the CNS restricted antigen MOG, have been tested in EAE. The CAR construct encoded an extracellular anti-MOG scFv, transmembrane CD3 domain, intracellular signaling domains of CD3ζ and CD28, internal ribosomal entry site (IRES), and the transcription factor *Foxp3* (36) to confer myelin-specificity and redirect T cells into the Treg lineage (36). Murine CD4^+^ T cells transfected with the MOG-CAR-FOXP3 construct were immunosuppressive and suppressed Tcons in coculture as well as ameliorated MOG_35-55_-induced EAE in C57BL/6 mice when administered therapeutically at peak of disease (36). Likewise, human Tregs transduced with a CAR encoding anti-MBP or anti-MOG scFv, transmembrane CD28 and the intracellular signaling domains of CD28 and CD3ζ, suppressed MOG_35-55_-induced EAE in C57BL/6 mice (227). Similarly, CD4^+^ CAR-Tregs recognizing 2,4,6 Trinitrophenol (TNP) suppressed TNP-induced colitis in mice, decreased colonoscopy colitis scores, and increased survival (**Table 6**) (228).

CD4^+^ CAR-Tregs have also been tested in a murine model of T1D. The CAR construct encoded an extracellular anti-insulin scFv, transmembrane CD8 domain, the intracellular signaling domains of CD28 and CD3ζ, T2A self-cleaving peptide, and the transcription factor *Foxp3* (37). The insulin-CAR-FOXP3-T cells were recruited into the Treg lineage and were activated and proliferated in response to aggregated insulin, but not monomeric insulin. Therefore, it was hypothesized that insulin-CAR-FOXP3-T cells would only be activated in the pancreas where aggregated insulin is secreted. Nonetheless, the insulin-CAR-FOXP3-T cells were unable to suppress the development of T1D when adoptively transferred into prediabetic NOD mice. Although the insulin-CAR-FOXP3-T cells lacked efficacy, these CAR-Tregs were long lived and could identified 4 months after adoptive transfer.

CD4^+^ CAR-Tregs are also being investigated as a strategy to suppress undesirable immune responses against recombinant protein-based therapeutics that result in the formation of neutralizing antibodies which inhibit therapeutic efficacy. For example, hemophilia A patients treated with Factor VIII (FVIII) replacement therapy often develop FVIII-neutralizing antibodies that abrogate therapeutic efficacy (248). Therefore, FVIII-CAR-Tregs were tested to prevent FVIII-specific immune responses in mice. The CAR construct encoded an extracellular anti-FVIII scFv, transmembrane CD28 domain, and the intracellular signaling domains of CD28 and CD3ζ (229). Human FVIII-CAR-Tregs exhibited antigen-specific suppression and prevented the proliferation of a FVIII-CAR-T cells, better than natural polyclonal Tregs, when cocultured with FVIII and PBMCs *in vitro*. Human FVIII-CAR-Tregs were tested in FVIII-knockout mice (E16) x humanized DR1 mice immunized with FVIII in incomplete Freund’s adjuvant and suppressed the development of FVIII-specific antibodies.

Furthermore, CD4^+^ CAR-Tregs have been investigated as an intervention for allergic disease. CEA-CAR-Tregs were tested in a murine model of OVA induced allergic airway inflammation in carcinoembryonic antigen (CEA) Tg mice that express CEA on the luminal surface of the pulmonary and the gastrointestinal tract epithelia (231). The CAR construct encoded an extracellular anti-CEA scFv, transmembrane CD4 domain, and the intracellular signaling domains of CD28 and CD3ζ. The CEA-CAR-Tregs homed to the lungs which expresses high levels of CEA in CEA-Tg mice. Following adoptive transfer to CEA-Tg mice, the CEA-CAR-Tregs reduced airways hyper-reactivity, inflammation, mucus production, and eosinophilia in experimental OVA induced asthma. Furthermore, CEA-CAR-Tregs reduced the production of proinflammatory cytokines IL-5 and IL-15 as well as prevented the accumulation of pathogenic IgE antibodies.

***CD8^+^ CAR-Tregs:*** Although the specific function of CD8^+^ Tregs remains to be determined, it was reported that CD8^+^ Tregs are immunosuppressive and contribute to immune tolerance in mice and human (249, 250). Human CD8^+^ A2-CAR-Tregs were activated, as seen with CD4^+^ A2-CAR-Tregs, in the presence of HLA-A2^+^ cells (234). The CD8^+^ A2-CAR-Tregs were not inherently cytotoxic as they did not kill HLA-A2^+^ cells or induce weight loss when adoptively transferred into in HLA-A2 transgenic NSG mice. Additionally, human CD8^+^ A2-CAR-Tregs prevented human HLA-A2^-^ T cell rejection of human allogenic HLA-A2^+^ skin grafts and xenograft GVHD induced with HLA-A2^+^ PBMCs in NSG mice.

***CAAR- and BAR-Tregs:*** Antigen-specificity can also be conferred to Tregs using a specific type of CAR receptor known as chimeric autoantigen receptor (CAAR) or B cell-targeting antibody receptor (BAR). These receptors express antigens that are recognized by autoreactive T or B cells, instead of the conventional extracellular antigen recognition domains such as scFv. CAAR- and BAR-Tregs are activated when antigen-specific B and T cell recognize their cognate antigens included in the CAAR/BAR construct and drive CAAR/BAR crosslinking. These receptors act as bait to trap and suppress/kill antigen-specific lymphocytes.

Antigen-specificity has been conferred to Tregs using CAAR constructs that encode peptide-MHCII complexes. For example, a CAAR construct encoded an extracellular MBP_89-101_-I-As chains fused to the intracellular signaling domain of CD3ζ (235). The I-As-MBP_89-101_ CAAR domain was designed to lure pathogenic MBP_89-101_-specific CD4^+^ T cells to I-As-MBP_89-101_ CAAR-Tregs for suppression. The CD4^+^ MBP_89-101_-I-As-CAAR-Tregs suppressed MBP_89-101_-induced EAE in SJL mice, when administered at the time of disease induction or at peak disease. Lymphocytes from MBP_89-101_-I-As-CAAR-Treg treated mice exhibited decreased antigen-specific T cell responses and increased IL-4 and IL-10 production. Monoclonal antibody mediated neutralization of IL-10 and IL-4 abrogated MBP_89-101_-I-As-CAAR-Tregs mediated suppression. Interestingly, T cells from MBP_89-101_-I-As-CAAR-Treg treated donor mice suppress MBP_89-101_-induced EAE in recipient mice even after depletion of MBP_89-101_-I-As-CAAR-Treg. Therefore MBP_89-101_-I-As-CAAR-Treg induced MBP_89-101_-specific suppressor cells that could prevent EAE.

BAR-Tregs are being investigated as a therapeutic for allergic disease and were tested in a murine model of anaphylaxis. The BAR construct encoded an OVA extracellular antigen domain, transmembrane CD28 domain, and intracellular CD28 and CD3ζ signaling domains (236). Upon adoptive transfer into OVA sensitized BLAB/c mice the CD4^+^ OVA-BAR-Tregs did not result in anaphylaxis, in response to the OVA contained in the BAR construct. Instead, murine or human OVA-BAR-Tregs suppressed anaphylaxis in OVA sensitized BALB/c mice when mice were re-challenged with OVA. The OVA-BAR-Tregs also suppressed anti-OVA IgE mediated mast cell induced anaphylaxis.

BAR-Tregs are also being investigated to prevent undesirable immune responses to recombinant therapeutic drugs. Human FVIII-BAR-Tregs blocked the formation of FVIII-specific antibodies when administered before FVIII sensitization and prevented the further development of FVIII-specific antibodies when administered between FVIII sensitizations in FVIII knockout x DR1 mice (237).

#### TCR-Treg Cell Therapy

The antigen specificity of polyclonal Tregs can be redirected using genetically engineered TCR. TCR-Tregs can recognize both extracellular and intracellular antigens in the contexts of MHCII while CAR-Tregs are limited to extracellular antigens. However, TCR-Tregs are restricted by MHCII/antigen availability as a well as MCHII haplotype.

TCR-Tregs are being tested to suppress organ-specific autoimmunity. For example, TCR-Tregs have been tested in a murine model of arthritis. The TCR constructs encoded OVA-reactive TCRα and β chains and in some cases included a *Foxp3* transcript (238). Murine CD4^+^ Tregs were transduced with the OVA-reactive TCR construct that lacked *Foxp3* while CD4^+^ T cells were transduced with the OVA-reactive TCR construct containing *Foxp3*. Expression of FOXP3 redirected T cells into the Treg lineage. Both OVA-TCR-Tregs and OVA-TCR-FOXP3-T cells elicited bystander suppression and suppressed a nucleoprotein-reactive CD8^+^ T cell line when cocultured with DCs, OVA, and nucleoprotein. The OVA-TCR Tregs and OVA-TCR-FOXP3-T cells suppressed methylated bovine serum albumin (mBSA)-induced arthritis in C57BL/6 mice and reduced knee swelling when mice were re-challenged with mBSA and OVA but not mBSA alone. However, OVA-TCR-FOXP3-T cells were less effective at suppressing disease compared to OVA-TCR-Tregs.

Additionally, myelin-specificity has been conferred to human Tregs *via* the expression of an MBP_85-99_-specific HLA-DR15-restircted TCR (239). The engineered MBP_85-99_-TCR-Tregs displayed antigen-specific suppression and prevented the proliferation of MBP_85-99_-specific effector T cells *in vitro*. Furthermore, the MBP_85-99_-TCR-Tregs were superior compared to OVA_323-339_-TCR Tregs at protecting HLA-DR15 transgenic mice from MOG_35-55_-induced EAE. Therefore, TCR-Tregs exhibit organ-specific bystander suppression. In addition, human polyclonal Tregs have been redirected to pancreatic antigens using islet-specific TCR (**Table 6**) (251).

In addition, human TCR-Tregs expressing a TCR-specific for FVIII were tested for their ability to block FVIII generated immune responses in FVIII knockout x DR1 humanized mice (240). The FVIII-TCR-Tregs were immunosuppressive and blocked FVIII-TCR-Tcon responses as well as the formation of FVIII-specific antibodies more efficiently than polyclonal Tregs *in vitro*.

#### CAR-T Cell Therapy for Autoimmune Diseases

***CAR-T cell therapy:*** Cytotoxic CD8^+^ CAR-T cells are being explored as a therapeutic strategy to deplete pathogenic lymphocytes involved in the etiology of autoimmune and allergic disease. CAR-T cells may have several advantages over mAb-based cell depletion strategies currently used to treat autoimmune disease, including anti-CD20 and anti-CD52 which deplete B cells and B/T cells, respectively (252, 253). First, CAR-T cells are long lived cells that can multiply, while antibodies are constrained by a pharmacological half-life and require repeated administrations to achieve and maintain therapeutic efficacy. Furthermore, CAR-T cells can traffic to the lymphoid tissues or target organs and develop into memory populations that can prevent the reemergence of pathogenic lymphocytes. Therefore, CAR-T cells therapy may only require a single dose to achieve lasting therapeutic efficacy. Second, CAR-T cell may have a higher potency than mAb therapies and may more efficiently delete pathogenic immune cells. These attributes suggest that CAR-T cell therapies may be an efficacious means to deplete pathogenic immune cells to alleviate autoimmunity. However, caution should be taken as CAR-T could produce massive amounts of inflammatory cytokines following activation, which could exacerbate autoimmunity.

CD8^+^ CAR-T cells are being designed to kill B cells that express the B cell restricted surface molecule CD19, as a potential therapeutic for SLE (241). CD8^+^ CD19-CAR-T cells eliminated B cells, reduced circulating IgM, IgG, and pathogenic anti-DNA antibodies as well as reduced the ratio of CD4/CD8^+^ T cells in murine models of SLE. A single dose of the CD19-CAR-T cells suppressed murine SLE when administered before or after the development of clinical disease in MRL-lpr and MZB/w mice, respectively. The CD19-CAR-T cells depleted B cells throughout the experiment which lasted approximately one year. Moreover, CD8^+^ T cells transferred from donor mice treated with CD19-CAR-T cell 7 months prior, depleted B cells in recipient mice. These results suggest that B cell depletion with CAR-T cells may provide long term protection in autoimmune diseases. In addition to CD19, the transmembrane IgE receptor expressed on B cells was explored as a B cell target for the CD8^+^ CAR-T cells (**Table 6** **)** (242).

APCs presenting autoantigens including the insulin beta chain peptide 9-23 (InsB_9-23_) which binds to the MHCII (I-A^g7^) in NOD mice can activate pathogenic insulin-specific T cells that drive T1D (254). Consistent with these findings, a mAb specific for InsB_9-23_-I-A^g7^ complex prevented diabetes in NOD mice by a mechanism that was hypothesized to be dependent on APC depletion (243, 254). Therefore, a CAR-T cell construct was designed to kill InsB_9-8_ presenting APCs. The CAR encoded anti-InsB_9-23_-I-A^g7^ scFv, transmembrane CD28 domain, and intracellular CD28 and CD3ζ signaling domains (243). A second-generation construct also contained the intracellular signaling domain of CD137 which increased CAR-T cell persistence *in vivo*. The CD8^+^ anti-InsB_9-23_-I-A^g7^-CAR-T cells were activated and killed cells that expressed InsB_9-23_-I-A^g7^ but not control I-A^g7^construct. Adoptively transferred anti-InsB_9-23_-I-A^g7^-CAR-T cell homed to the pancreatic lymph nodes and delay the development of T1D by approximately 5 weeks.

***CAAR- and BAR-T cell therapy:*** Cytotoxic T cells have been engineered to express a CAAR construct encoding the peptide-MCHI complex that autoreactive CD8 T cell recognizes. For example, a mRNA based CAAR construct encoding InsB peptide 15-23 (InsB_15-23_) fused to the extracellular β2m and intracellular CD3ζ signaling domain redirected CD8^+^ T cells against pathogenic insulin-specific CD8^+^ T cells that recognize the MHCI/InsB_15-23_ complexes (244). The InsB_15-23_-β2m-CAAR construct killed InsB_15-23_-specific CD8^+^ T cell hybridoma line. When adoptively transferred into prediabetic 5-6-week-old NOD mice, the CD8^+^ InsB_15-23_-β2m-CAAR-T cells reduced CD4^+^ and CD8^+^ T cell infiltration into pancreatic islets and protected NOD mice from the development of T1D.

Likewise, antigen-specificity has also been conferred to T cells using CAAR constructs that encode antigen domains. For example, CAAR-T cells that express extracellular Desmoglein 3 (DSG3) domains were designed to deplete DSG3-specific B cells, which drive the pathogenesis of PV. A CAAR-T cell construct was designed that encoded an extracellular DSG3 domain, transmembrane CD8α domain, and the intracellular signaling domains of CD137 and CD3ζ (245). Human DSG3-CAAR-T cells produced IFN-γ and specifically lysed a DSG3-specific hybridoma cells even in the presence of soluble DSG3-specific antibodies that could potentially block the CAAR extracellular DSG3 domain. Likewise, human DSG3-CAAR-T cell eliminated DSG3-specific B cells in humanized NSG mice. The DSG3-CAAR-T cells did not exhibit off target cytotoxicity and prevented DSG3-specific hybridoma driven GVHD in NSG mice. In addition, CAAR-T cells that express extracellular muscle-specific receptor kinase (MuSK) domains were designed to delete pathogenic MuSK-specific B cells, that drive MG **(** **Table 6** **)** (246).

These preclinical studies led to the initiation of 3 clinical trials using CAR- or CAAR-T cells to deplete pathogenic B cells in MG, PV, and SLE (**Table 1**). For MG, a phase I/II clinical trial has been devised to test the safety and efficacy of anti-B-cell maturation antigen (BCMA)-CAR-T cells designed to deplete activated B cells (NCT04146051). For PV, a phase I clinical trial has been devised to test the safety of a DSG3-CAAR-T cells designed to deplete DSG3-specific autoreactive B cells (NCT04422912). Finally, for SLE, a phase I clinical trial will test the safety of CD19-CAR-T cells designed to deplete B cells (NCT03030976). In addition, CD19-CAR-T cells will be tested with or without an intracellular signaling CD137 domain (255).

## IL2-Mediated Therapy

Differentiation, function, and maintenance of Tregs are dependent on IL-2. In mouse models, deficiency of IL-2 or its receptor components leads to spontaneous autoimmune phenotype due to defects in the generation of functional Tregs (256–259). In humans, allelic variants associated with reduced expression of IL-2 or its receptor chains, or genes that impact the IL-2 receptor signaling pathway, have been associated with increased risks of multiple autoimmune and inflammatory diseases, including T1D, SLE, RA, and MS (260–266). IL-2 drives proliferation of Tregs and enhances expression of FOXP3 and additional genes that define Treg cell phenotype and function [review by (267, 268)]. These effects of IL-2 can be recapitulated by expression of a constitutively activated form of STAT5 in mouse Tregs (269), further highlighting the role of the IL-2 receptor-JAK/STAT signaling pathway in these cells. Additionally, although not required, IL-2 can induce proliferation and activation of other cell types including FOXP3^─^ T cells (Teff/Tcon), NK cells, and ILC2 (type 2 innate lymphoid cells) (268).

### Low Dose IL-2 Therapy

Recombinant IL-2 at high doses is an approved therapy for the treatment of advanced and metastatic cancer [review by (270)]. In high dose IL-2 therapy, efficacy is driven by the ability of IL-2 to enhance conventional and effector T and NK cell responses against tumor cells, but the therapeutic window is limited by significant and often severe toxicity. In contrast, evidence from over a decade of research has indicated that Tregs not only require IL-2 for function and stability, but that they are exquisitely sensitive to IL-2 (271, 272). Tregs constitutively express high levels of CD25 (IL2Rα) (273), which captures IL-2 and is assembled with CD122 (IL2Rβ) and the common γ chain CD132 (IL2Rγ), into a high-affinity heterotrimeric receptor complex. As a result, Tregs exhibit increased affinity to IL-2 compared to other cell types that express lower levels of or lack CD25. Increased affinity to IL-2 allows Tregs to compete and “soak up” free IL-2 (274). Moreover, Tregs can proliferate in response to limiting amounts of IL-2 compared to non-Treg cells *in vivo* (275). These findings have formed the basis of the low-dose (LD) IL-2 therapy for treatment of autoimmune and inflammatory diseases, where limiting amounts of recombinant IL-2 preferentially expand and enhance function of Tregs which in turn suppress pathogenic inflammatory responses.

Data emerging from multiple clinical studies support the therapeutic rationale for LD IL-2 therapy. For example, LD IL-2 therapy ranging from 1.0x10^6^ IU/day (276), 1.5x10^6^ IU/day (277), or 1.0x10^6^ IU every other day (278) led to 2-5 fold expansion of Tregs over baseline in HCV-induced vasculitis (277), chronic GVHD (275, 276), and SLE (278, 279) patients, which appeared to correlate with disease improvement in a subset of patients. Nonetheless, dose-limiting toxicity (DLT) was observed, which confined the therapeutic dose range of IL-2 to a narrow 2-3-fold window. Specifically, significant toxicity was observed at 3.0x10^6^ IU/day which required dose reduction to 1.5x10^6^ IU/day (280) and similarly 3.0x10^6^ IU/m^2^/day was reduced to 1x10^6^ IU/m^2^/day (281). As a potential explanation for the observed toxicity, significant Tcon/Teff and NK cell expansion was observed, in some cases greater than that observed for Tregs (276, 281). With better understanding of safe dose range that induces Treg expansion in disease patients, efficacy of low LD IL-2 either alone or in combination therapies continues to be evaluated in a broader range of indications in multiple ongoing phase II clinical trials (**Table 7**) (291, 292).

**Table 7:** Summary of IL-2 therapies in development.

	Format/MOA of Treg selectivity	Current status	References
LD IL-2 therapy	Low dose (below 3x10^6 IU/day or 3x10^6 IU/m2/day) of recombinant IL-2 (Proleukin); limiting amounts of IL-2 preferentially acts on CD25hi Tregs	Ph2 Ph1	Active Ph2 studies: NCT04065672 (Behcet’s Disease) NCT03776643 (Allergy) NCT04263831 (CD) NCT01988506 (TRANSREG)
IL-2 mutein therapy * AMG592 = Efavaleukin Alpha * PT-101	Attenuated IL-2 with HLE; weaker affinity enhances selectivity for CD25hi Tregs	Ph2 (AMG592 = Efavaleukin alpha) Ph1a (PT-101)	NCT03451422 NCT03422627 NCT04680637 Pandion news announcement Jan 4, 2021 (PT-101)
PEGylated IL-2 * NKTR-358= LY3471851	Multiple PEG moieties attenuate IL-2 affinity to receptor subunits; weaker affinity enhances selectivity for CD25hi Tregs	Ph2 Ph1	Ph2: NCT04433585 (SLE) NCT04677179 (UC) Ph1: NCT04081350 (Eczema) NCT04119557 (Psoriasis)
IL-2 mutein engineered receptor signaling clamp	IL-2 mutein with enhanced affinity to CD122 and reduced affinity to CD132; partial agonist that blocks IL-2 receptor binding to endogenous IL-2, reduces activation of pathogenic cells such as Teff and NK cells	Preclinical	(282)
IL-2cx	IL-2:IL-2 Ab complex modifies IL-2 conformation to bias interaction with CD25 and attenuates binding	Preclinical	(283–286)
CD25-IL2 fusion	Multimeric intermolecular complexes between CD25 and IL-2 limit IL-2 interaction with cell surface receptors	Preclinical	(287, 288)
IL2 dual functional molecules	IL233; IL2-TNFR2 agonist Combinatorial and synergistic stimulation of IL-2 and Treg-biased pathways enhances Treg selective effects	Preclinical	(289, 290)

### Engineered IL-2 Therapy

Results from the LD IL-2 therapy have encouraged subsequent development of approaches to increase the Treg-selective effects of IL-2 while reducing potentially disease-exacerbating effects of non-Treg cells, to achieve a wider therapeutic dose range. Engineered versions of IL-2 to modify the activity and binding specificity to Treg versus non-Tregs and IL-2 complexed with anti-IL-2 antibody that indirectly impacts activity and selectivity to the high affinity IL2 receptor are among these approaches. In most cases, these approaches also include a modification to extend the *in vivo* half-life of IL-2.

Several examples of engineered human and murine IL-2 molecules harboring mutations that attenuate interactions with the IL-2R complex have been described as IL-2 muteins. Among these, IL-2 muteins that decrease interactions with the signaling components of the IL2R complex, namely CD122 and CD132, but retain affinity to CD25 have been reported and are currently being tested in clinical trials for autoimmune and inflammatory diseases (**Table 7**). Due to the mutations that inhibit interaction between IL-2 and CD122/CD132, these IL-2 muteins induced weaker STAT5 activation compared to wildtype IL-2 (WT). However, because Tregs express high affinity trimeric IL2R, IL-2 muteins had activity that was less diminished in Tregs than non-Tregs, resulting in enhanced Treg selectivity. Fused to full IgG (293) or the Fc portion of IgG (294, 295). human IL-2 muteins showed prolonged *in vivo* half-life compared to recombinant human IL-2 (Proleukin) and induced preferential expansion of Tregs over CD4^+^ Teff or NK cells *in vivo* in non-human primates (293) and in humanized mouse models (296). Data from the first-in-human trial for IL-2 mutein (AMG 592 “Efavaleukin alpha”) supports the rationale for this approach in human subjects, where Treg expansion was observed to a similar degree as the LD IL-2 therapy, but the expansion of non-Tregs (particularly Tcon and NK cells) (281, 297) was significantly reduced (295). In parallel, mouse IL-2 muteins with enhanced Treg selectivity and *in vivo* half-life, expanded highly suppressive Tregs in tissues (298) and controlled autoimmunity in the mouse NOD T1D model (299). Moreover, the IL-2 mutein prevented antibody response to FVIII in a mouse model of hemophilia A (300), further supporting that Tregs responding to an attenuated IL-2 mutein exert immune suppressive effects. Another class of IL-2 muteins with enhanced affinity to CD25 has also been described, although the activity and selectivity profile of such muteins need to be further evaluated (294).

Since endogenous WT IL-2 also activates non-Tregs, the *in vivo* efficacy of IL-2 muteins in inflammatory settings is likely to be influenced by the combined effects on Tregs and IL-2-sensitive non-Tregs that might be pathogenic, rather than strictly by its effects on Tregs alone. In fact, an antagonist IL-2 mutein with enhanced binding to IL2Rβ chain but reduced signaling capacity reduced inflammatory response in a mouse GVHD model (282), presumably by displacing and antagonizing the effects of endogenous WT IL-2 on pathogenic cells such as Teff and NK cells. The mechanism of action of this class of “engineered receptor signaling clamp” IL-2 muteins is distinct from the above-mentioned IL-2 muteins that preferentially act on Tregs by retaining affinity to CD25 and delivering agonist activity.

In addition to Teff and NK cells, ILC2 and T follicular helper (T_FH_) cells are particularly relevant targets of IL-2 and may impact disease activity. ILC2s express high levels of CD25, produce IL-5 and induce eosinophil expansion in response to the LD IL-2 therapy (301). Consistently, multiple LD IL-2 clinical studies reported transient treatment-related increases in serum IL-5 levels and eosinophil numbers (276, 280, 292). Additionally, IL-2 is thought to inhibit T_FH_ cell differentiation during T cell priming (302) which may reduce autoantibody production. Interestingly, LD IL-2 suppressed the number of T_FH_ cells in SLE patients (278), suggesting that limiting amounts of IL-2 was sufficient to inhibit the T_FH_ cell differentiation.

Characterization of a panel of human IL-2 muteins that had activity spanning a broad spectrum of STAT5 activation signaling demonstrated that attenuation of IL-2 signaling asymmetrically impacted Treg and non-Treg cell responses (296). As the affinity of IL-2 to its receptor declined, STAT5 signal in non-Tregs decreased first, while it was maintained over a wider range of attenuation in Tregs. Different sensitivity of Treg versus non-Tregs toward IL-2 was partly but not entirely due to the differences in CD25 levels, and additional cell-intrinsic mechanisms are likely to contribute (272). If attenuated IL-2 is inherently better at maintaining Treg responses, it also explains why engineering or modifications that weakens the IL-2 and its receptor interaction *via* steric hindrance enhances Treg selectivity. These include PEGylated IL-2 (NKTR-358 “LY3471851”) (303), which induced clinical response and dose-dependent changes in Treg functional markers in a phase I trial in mild-to-moderate SLE, various IL-2:IL-2 Ab complexes described as CD25-directed IL-2cx (283, 284) and CD25-IL2 fusion proteins (287, 288). Anti-IL-2 antibodies that form complex with IL-2 can attenuate the IL2R signal by sterically inhibiting the IL-2:IL2R interactions but they can also modify the IL-2 structure to influence the bias with which it interacts with the receptor subunits (285, 286). Likewise, CD25-IL-2 fusion proteins formed multimers *via* intermolecular binding, effectively limiting the amount of free IL-2 that could bind to CD25 on cell surfaces. While the concept behind attenuated IL-2 muteins is similar to that of the LD IL-2 therapy, the intended advantage of the engineered attenuated IL-2 is the increased dose range where Treg-to-non-Treg selectivity is safely enhanced.

The next generation of IL-2-based modality is likely to include tissue- and/or Treg-directed and combination cytokine approaches. It may be possible to target IL-2 to inflamed tissue sites *via* tissue-specific or inflammation-specific markers. The most challenging task to this approach is identification of suitable markers for targeting, and to maintain Treg-to-non-Treg selectivity when delivering IL-2 to sites where both cell types reside in close contact. Recently, a hybrid cytokine that can simultaneously engage IL-2 and IL-33 receptors (IL233) expanded Treg and ILC2 and conferred protective effects in an animal model of kidney injury (289). Likewise, a dimeric dual-acting cytokine containing both IL-2 and TNF receptor 2 (TNFR2) agonist enhanced Treg expansion and phenotype better than either IL-2 or TNFR2 agonist alone *in vitro* (290). Although the *in vivo* activity and selectivity of this molecule was not evaluated, it presents an interesting possibility of simultaneously engaging synergistic pathways for more robust or selective induction of Treg responses.

## Concluding Remarks

Therapeutics are emerging with the aspiration to achieve lasting immune tolerance and provide a cure by addressing the root cause of disease. In this review, we covered three areas of emerging therapeutics which induce immune tolerance. How knowledge gained from the bench will be translated into bedside remains to be seen. It is critical to note that there is no tolerogenic vaccine nor T cell therapy approved for the treatment of autoimmune disease as of today. The advancement of tolerogenic vaccines has been difficult due to a lack of understanding of which mechanism of action is the most effective to achieve immune tolerance, shortage of biomarkers, and complexities within the pathogenesis of individual autoimmune diseases. Equipped with new technology platforms and growing knowledge of the autoantigen repertoire that drives specific autoimmune diseases, the next wave of emerging tolerogenic vaccines hold promises to overcome these obstacles. When considering a tolerogenic vaccine for the treatment of autoimmune disease, researchers should investigate the dose, administration route, and epitope components for tolerogenic vaccines as these components affect the immunological outcomes. Tolerogenic adjuvants can be used to augment the local environment and favor tolerance under proinflammatory conditions. However, special care must be taken to address whether these tolerogenic adjuvants induce systemic immune suppression or engender immune tolerance. Current evidence suggests that DC, LSEC, and marginal zone macrophage encompass a group of specialized APCs that favor tolerance *via* the induction of Tregs or deletion of autoreactive T cells. An ideal tolerogenic vaccine should induce organ-specific tolerance which can restrain autoantigen responses beyond those included within the tolerogenic vaccine. Finally, while most tolerogenic vaccines tested in the clinic have been safe, caution is required, as these vaccine platforms have the potential to activate autoantigen-specific T effector and B cell responses thereby exacerbating disease or even resulting in IgG-induced anaphylactic shock. Thus, it is critical to determine the best therapeutic window at which a therapeutic achieves the greatest benefit without resulting in unacceptable side-effects or toxicity. For example, recombinant WT IL-2 therapy is restrained to a narrow “low dose” therapeutic window due to dose-limiting toxicities. Likewise, one must consider the timing at which to stage a therapeutic intervention. For example, tolerogenic vaccines may be more effective when administered during early disease when epitope spreading is minimal or during disease remissions when clinical disease is suppressed. Careful design of clinical trials, especially for the phase I studies along with the development of clinical biomarkers associated with therapeutic benefit will likely determine the success of future clinical trials.

Although promising, there are still many barriers to overcome for the practical use of T cell therapies. For therapies using Tregs as a drug product, considerable efforts will be needed to establish a process to manufacture engineered Tregs within reasonable costs to benefit broad patient populations. The production of suitable quantities of functional, stable, and pure Tregs would be the most crucial step. Unlike CD8 or even CD4 T cell counterparts used in CAR-T cell therapies with greater proliferative potentials, it is difficult to expand Tregs. Moreover, there is no Treg-exclusive markers to confirm the purity of the Treg product. For example, CD25 and FOXP3 are used as markers for Tregs, however these markers are also transiently expressed by proinflammatory pathogenic T cells. Thus, standard protocols that ensure the quality of Treg cell therapy drug needs to be established. Furthermore, the transfer of high numbers of Tregs can lead to systematic immunosuppression similar to that of immunosuppressive agents. For T cell therapies using engineered cytotoxic T cells as a drug product, how transferred T cells behave under inflammatory environments associated with autoimmune disease is unclear. CAR/CAAR/BAR T cells produce multiple arrays of inflammatory cytokines which may exacerbate autoimmune disease. Finally, it remains to be determined which therapies discussed can establish long-lasting tolerance. To establish long-lasting tolerance, the treatment will likely need to induce long-lived memory Tregs or suppressor cells specific to disease-relevant antigens or induce differentiation of memory T cells that continue to kill pathogenic autoreactive cells. Additionally, it is unclear if therapeutics which are antigen independent such as IL-2 therapies which expand polyclonal Tregs or broad immune cell depletion (e.g. CD19^+^ B cell depletion) that rebalance or reset the immune system can lead to lasting immune tolerance.

In this review, we explored three areas of therapeutics that aim to achieve immune tolerance. The breadth of the therapeutic modalities designed to mediate immune tolerance continues to grow. We are excited for the future of immune tolerance therapies and the potential benefits they bring to alleviate patient suffering.

## Author Contributions

All authors contributed to the article and approved the submitted version. CM, SS, and HP wrote the manuscript and approved it for publication. HP edited and finalized the manuscript.

## Conflict of Interest

CM, SS, and HP are employees of Amgen Inc.

## Abbreviations

AAV, Adeno-Associated Virus; AHR, Aryl Hydrocarbon Receptor; APC, Antigen Presenting Cell; BAR, B Cell-Targeting Antibody Receptor; BTK, Bruton’s Tyrosine Kinase; CAAR, Chimeric Autoantigen Receptor; CAR, Chimeric Antigen Receptor; CD, Cluster of Differentiation; CEA, Carcinoembryonic Antigen; CNS, Central Nervous System; CTLA4, Cytotoxic T-Lymphocyte Associated Protein-4; CYP2D6, Cytochrome P450; DC, Dendritic Cell; DCIR2, DC Immunoreceptor 2; DC-ASGR, DC-Asialoglycoprotein Receptor; DLT, Dose-Limiting Toxicity; DNA, Deoxyribonucleic Acid; DTH, Delayed-Type Hypersensitivity; DSG3, Desmoglein 3; EAE, Experimental Autoimmune Encephalomyelitis; FOXP3, Forkhead Box Protein P3; FVIII, Factor VIII; GARP, Glycoprotein A Repetitions Predominant; GITR, Glucocorticoid-Induced TNFR-Related Protein; GM-CSF, Granulocyte Macrophage-Colony Stimulating Factor; GVHD, Graft Versus Host Disease; HLA, Human Leukocyte Antigen; ICOSL, Inducible T Cell Costimulator Ligand; IDO, Indoleamine 2,3-dioxygenase; ILC2, Type 2 Innate Lymphoid Cells; IL, Interleukin; IP, Intraperitoneal; IRAK4, Interleukin 1 Receptor Associated Kinase 4; IRES, Internal Ribosomal Entry Site; iTr35, inducible IL-35 producing regulatory T cells; ITE, 2-(1’ H-indole-3’-carbonyl)-thiazole-4-carboxylic acid methyl ester; IV, Intravenous; JAK, Janus Kinase; LAG3, Lymphocyte Activating Protein 3; LAP, Latency-associated peptide; LD, Low Dose; LSEC, Liver Sinusoidal Endothelial Cells; mAb, Monoclonal Antibody; MBP, Myelin Basic Protein; Mbsa, Methylated Bovine Serum Albumin; M-CSF, Macrophage Colony-Stimulating Factor; MG, Myasthenia Gravis; MHC, Major Histocompatibility Complex; MOG, Myelin Oligodendrocyte Glycoprotein; MS, Multiple Sclerosis; MuSK, Muscle-Specific Receptor Kinase; MP, Microparticle; NF-_K_B, Nuclear Factor Kappa B; NK, Natural Killer Cell; NO, Nitric Oxide; NP, Nanoparticle; NSG, NOD Scid Gamma mice; OVA, Ovalbumin; PBMC, Peripheral Blood Mononuclear Cell; PDC, Pyruvate Dehydrogenase Complex; PDL1, Programmed Cell Death 1 Ligand; PD1, Programmed Cell Death 1; PEG, Polyethylene Glycol; PGE-2, Prostaglandin E2; PLGA, Poly(lactic-co-glycolic acid); PLP, Proteolipid Protein; PV, Pemphigus Vulgaris; RA, Rheumatoid Arthritis; RBC, Red Blood Cell; RIP, Receptor-Interacting Protein; RNA, Ribonucleic Acid; SC, Subcutaneous; scFv, Single Chain Variable Fragment; SLE, Systemic Lupus Erythematosus; SYK, Spleen Associated Tyrosine Kinase; T1D, Type 1 Diabetes; Tcon, T Conventional Cell; TCR, T Cell Receptor; tDC, Tolerogenic Dendric Cell; Teff, T Effector Cell; TGF-β, Transforming Growth Factor-beta; Th cell, T Helper Cell; TIGIT, T Cell Immunoreceptor With Ig And ITIM Domains; TIM-3, T Cell Immunoglobulin Mucin 3; TLR, Toll Like Receptor; TNF-α, Tumor Necrosis Factor-alpha; TNFR2, TNF Receptor 2; TNP, 2,4,6 Trinitrophenol; TPL2, Tumor Progression Locus 2; TRAIL, TNF-Related Apoptosis-Inducing Ligand; Treg, T Regulatory Cell; Tr1, Type 1 Regulatory T Cell; TYK2, Tyrosine Kinase 2.
